# TBX1 targets the miR-200–ZEB2 axis to induce epithelial differentiation and inhibit stem cell properties

**DOI:** 10.1038/s41598-022-24604-9

**Published:** 2022-11-23

**Authors:** Noriko Funato, Hiromi Yanagisawa

**Affiliations:** 1 Department of Signal Gene Regulation, Tokyo Medical and Dental University (TMDU), Tokyo, 113-8510 Japan; 2 Research Core, Tokyo Medical and Dental University (TMDU), Tokyo, 113-8510 Japan; 3 Life Science Center for Survival Dynamics, Tsukuba Advanced Research Alliance, University of Tsukuba, Ibaraki, 305-8577 Japan

**Keywords:** Cancer, Cell biology, Developmental biology, Molecular biology

## Abstract

*TBX1*, which encodes a T-box transcription factor, is considered a candidate gene for DiGeorge syndrome, velocardiofacial syndrome, and conotruncal anomaly face syndrome. Transduction of TBX1 decreases cell proliferation in epithelial cancer cells and *Tbx1* ablation induces epithelial proliferation during palatal development. Here, we report that TBX1 regulates stem cell properties and epithelial differentiation through the transcriptional activation of microRNAs. Stable expression of TBX1 induces microRNA-200 (miR-200), whose members repress the epithelial-to-mesenchymal transition and induce epithelial differentiation. TBX1 rescues ZEB2-dependent transcriptional inhibition of the *miR-200b/200a/429* cluster, whose promoter region contains conserved overlapping *cis*-regulatory motifs of the ZEB-binding E-box and TBX-binding element. Consequently, TBX1 activates the expression of both miR-200 and stemness-inhibitor miR-203 to inhibit their common targets, *BMI1* and *ZEB2*. Moreover, *Tbx1* ablation affects the differentiation of the palatal epithelium and perturbs the expression of miR-200, miR-203, and their target genes. We propose that TBX1 links stem cell properties and epithelial differentiation by inducing miR-200 and miR-203. Thus, targeting of the ZEB2–miR-200 axis by TBX1 may have potential therapeutic implications in miR-200-associated tumors and cleft palate.

## Introduction

*TBX1*, which encodes a T-box transcription factor, is considered a candidate gene for DiGeorge syndrome (OMIM #188,400), velocardiofacial syndrome (OMIM #192,430), and conotruncal anomaly face syndrome (OMIM #217,095)^1–3^. *Tbx1*-null (*Tbx1*^*Δ/Δ*^) mice exhibit craniofacial and cardiovascular phenotypes, including cleft palate phenotypes, in patients with DiGeorge syndrome^4–7^. *Tbx1* is transiently expressed in the undifferentiated oral epithelium, and its expression decreases upon subsequent palatal fusion^4^. *Tbx1* ablation increases the number of basal cells in the palatal epithelium and induces abnormal oral epithelial differentiation and intraoral epithelial adhesion^4^. In addition, TBX1 reduces cell growth in epithelial cancer cell lines^4,8^. These findings suggest that TBX1 is linked to both tissue morphogenesis and tumor growth.

The epithelial-to-mesenchymal transition (EMT) is critically important for the development and progression of carcinomas or disease conditions and is linked to the gain of stem cell properties^9,10^. EMT is associated with marked changes in cell–cell adhesion, polarity, motility, and migration, suggesting that EMT drives epithelial tumor invasion, embryonic development, and tissue remodeling^9,10^. EMT is typically characterized by the downregulation of the epithelial marker E-cadherin/CDH1 and upregulation of the mesenchymal marker N-cadherin/CDH2^9–11^. The EMT program is regulated by multiple networks of microRNAs (miRNAs) and EMT transcription factors, including the ZEB (ZEB1 and ZEB2/SIP1/ZFHX1B), Twist, and Snail families, whose expression and functional importance depend on the tissue type^10^.

The miR-200 family (miR-200) induces epithelial differentiation, thereby suppressing EMT in epithelial cancers, and is associated with reduced invasion, metastasis, and embryonic development^10^. miR-200 has five members derived from two microRNA clusters: *miR-200b/200a/429* are encoded on human chromosome 1p36.33 and *miR-141/200c* on chromosome 12p13.31. miR-200 inhibits mRNA translation of the EMT-activators ZEB1 and ZEB2, which harbor conserved miR-200 sites in their 3′ untranslated region (3′UTR)^12,13^. Conversely, miR-200 expression can be directly suppressed by ZEB2 and ZEB1, indicating that miR-200 and ZEB proteins reciprocally regulate each other via a feedback loop^12,14–16^. *Zeb1* ablation induces cleft palate in 50% of mice^17^, and embryos having the compound genotype of *Zeb1*- and *Zeb2*-deficient mice exhibit midfacial cleft, suggesting that ZEB1 and ZEB2 interact synergistically in midfacial development^18^. miR-200b, which is expressed in the palatal epithelium, represses the expression of *Zeb1* and *Zeb2* during palatogenesis^19^. Moreover, miR-200b targets angiogenesis-related genes, vascular endothelial growth factor-A (*VEGFA*) and its receptors *FLT1*/*VEGFR1* and *KDR*/*VEGFR2*/*FLK*^20,21^. VEGF signaling plays a crucial role in pathological and physiological angiogenesis, contributing to tumor progression from dormant in situ lesion to metastasis^22^. Along with miR-200, ZEB1 promotes tumorigenicity by repressing the stemness-inhibiting, skin-specific miR-203, which induces differentiation of skin stem cells into suprabasal cells^23,24^. Tumor suppressor miR-203 expression was enriched in keratin-high cells from cervical squamous cancers^25^. Despite the importance of EMT- and stemness-inhibiting microRNAs, a limited number of transcription factors have been reported to induce their expression, and the regulators that contribute to mesenchymal-to-epithelial transition (MET) remain to be elucidated.

Here, we investigated the role of the relationship among TBX1, EMT-inhibiting miRNAs, and the EMT-activator ZEB2 in the control of stemness. We found that TBX1 inhibited stem cell properties in cervical carcinoma cells by regulating the expression of miR-200, miR-203, and their target genes. Moreover, *Tbx1* ablation in mice inhibited epithelial differentiation, accompanied by the reduction of miR-200 and miR-203 and the induction of their target genes.

## Results

### TBX1 is a regulator of epithelial differentiation and stemness

**Figure 1:**
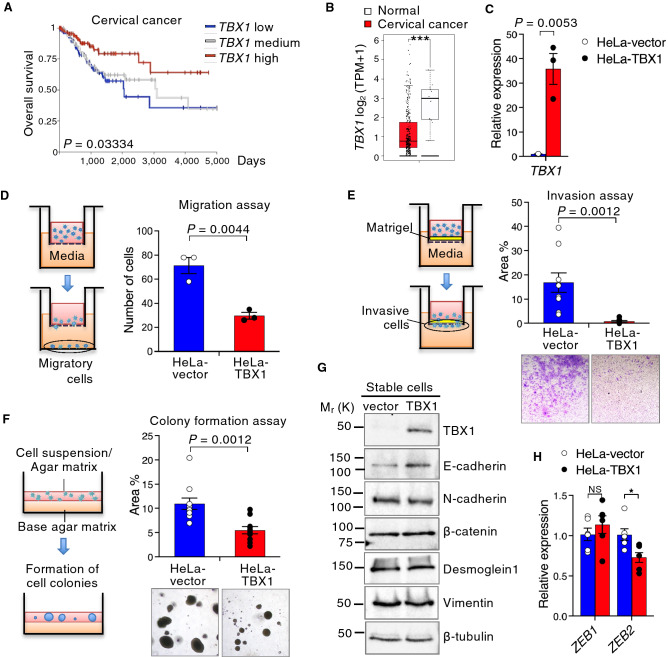
TBX1 inhibits cell migration, invasion, and soft agar colony formation. (**A**) Kaplan–Meier curve representing overall survival in patients with primary cervical cancer (*n* = 288) with high, medium, or low *TBX1* expression. Two-sided *P-*values were computed using a log-rank (Mantel-Cox) test. (**B**) Box plots were obtained from the GEPIA using the GTEx database to compare the expression of *TBX1* in cervical cancer (*n* = 306) and matched normal specimens (*n* = 13). TPM, transcripts per million. ***P ≤ 0.001; one-way ANOVA. (**C**) Expression of *TBX1* was examined by qPCR in HeLa cells stably transfected with control pEBMulti plasmid (vector) or pEBMulti-TBX1 plasmid (TBX1). *n* = 6. (**D**) Comparison of the relative migration of HeLa cells stably transfected with empty vector (HeLa-vector) or TBX1 (HeLa-TBX1) towards a 10% serum gradient for 24 h. *n* = 3. (**E**) In vitro invasion assays for HeLa cells stably transfected with empty vector or TBX1. Representative images of invaded cells are shown below the bar plot. *n* = 9. (**F**) Soft agar colony formation assays with HeLa cells stably transfected with the empty vector or TBX1. Colony-forming efficiency was determined after culturing for 3 weeks in the presence of hygromycin in three independent assays (*n* = 10). Representative images of colonies are shown below the bar plot. (**G**) Western blot analysis of extracts derived from HeLa cells stably transfected with empty vector or TBX1 for EMT-related markers. The full-length blots are shown in Supplementary data, Fig. S13. (**H**) Expression of *ZEB1* and *ZEB2* was examined with qPCR in HeLa-vector or HeLa-TBX1 cells (*n* = 6, *P ≤ 0.05; NS, not significant). For (**C-F**) and (**H**), the results are presented as mean ± s.e.m.; unpaired two-tailed Student’s *t*-test.

*Tbx1* ablation induces epithelial proliferation during palatal development, and transduction of TBX1 decreases cell proliferation in epithelial cancer cells^4,8^. Based on these findings, we speculated that TBX1 not only inhibits epithelial proliferation, but also affects tumorigenesis. To examine the role of TBX1 in epithelial carcinoma, we first investigated *TBX1* expression in carcinoma using human tumor bulk RNA-sequencing data from different origins. Stratification of patients by *TBX1* expression revealed increased overall survival for cervical cancer patients with higher *TBX1* expression (Fig. 1A). In contrast, no significant correlation was observed in head, neck, and lung squamous cell carcinomas (Supplementary data, Fig. S1A). Interestingly, *TBX1* expression was significantly lower in cervical cancer specimens than in matched normal tissues, which was different from the case in other squamous cell carcinomas (Fig. 1B and Supplementary data, Fig. S1B). Therefore, we speculated that TBX1 might be associated with EMT phenotypes in cervical cancer. Stable expression of TBX1 in HeLa cells, a cervical cancer cell line, induced strong upregulation of *TBX1* levels (Fig. 1C). Using control (HeLa-vector) and HeLa-TBX1 stably transfected cells, we investigated whether TBX1 contributes to the inhibition of invasive capacity and anchorage-independent cell growth. TBX1 inhibited cell migration (Fig. 1D), invasive capacity (Fig. 1E), and colony-forming capacity (Fig. 1F) compared to controls.

To analyze whether TBX1 overexpression alters the expression of EMT-related markers, we analyzed expression of the epithelial markers E-cadherin (CDH1), *β*-catenin, and Desmoglein1 (DSG1) as well as the mesenchymal markers vimentin and N-cadherin (CDH2). Stable expression of TBX1 increased the expression of E-cadherin, a master regulator of epithelial polarity and EMT in epithelial cancers^26^, whereas the levels of N-cadherin, *β*-catenin, Desmoglein1, and vimentin were unaffected (Fig. 1G). Analysis of cervical cancer and normal tissue data in The Cancer Genome Atlas (TCGA) database revealed a statistically significant correlation between *TBX1* and E-cadherin gene *CDH1*, whereas there was no correlation between *TBX1* and *CDH2* expression (Supplementary data, Fig. S2). These findings suggest that TBX1 overexpression partially reverts the mesenchymal phenotype to the epithelial phenotype in cervical cancer cells. ZEB proteins induce EMT by binding to E-boxes within the *CDH1* promoter and repressing its transcription^26^. Since TBX1 markedly reduced invasive capacity, accompanied by the reduced expression of E-cadherin/CDH1 (Fig. 1E–G), we speculated that the expression of TBX1 might be inversely correlated with that of EMT-related ZEB transcription factors. To address this possibility, we examined the expression of *ZEB1* and *ZEB2* in HeLa cells stably transfected with TBX1. *ZEB2* expression was significantly reduced in TBX1-transfected HeLa cells relative to that in the control cells (Fig. 1H), which suggested that TBX1 may be involved in maintaining the epithelial phenotype by inhibiting *ZEB2* expression.

**Figure 2:**
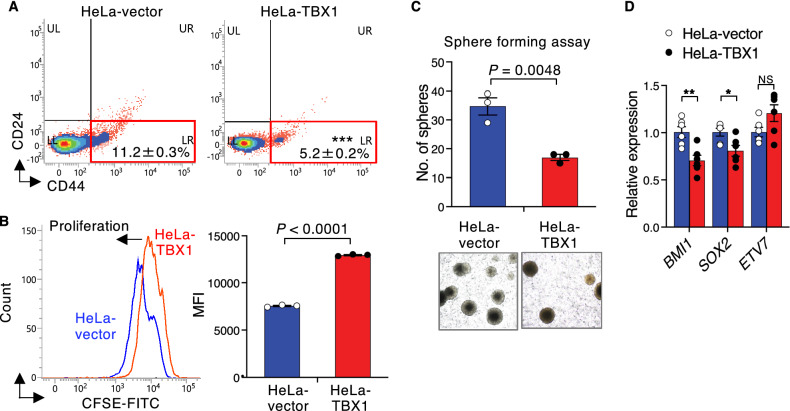
TBX1 suppresses stem cell properties. (**A**) Flow cytometric analysis of CD24 and CD44 expression in empty vector (HeLa-vector) and TBX1 stably transfected HeLa cells (HeLa-TBX1). The percentage of CD44^+^CD24^−^ stem cell population (lower right box) is indicated (*n* = 6). (**B**) Flow cytometric analysis of HeLa‑vector and HeLa‑TBX1 cells labeled with CFSE fluorescent dye. A representative day 2 histogram (left) and the mean ± s.e.m. of quantified results from three independent experiments with replicate wells (right) are shown (see also Supplementary data, Fig. S3A). MFI, mean fluorescence intensity. (**C**) Sphere-forming capacity of HeLa-vector and HeLa-TBX1 cells (*n* = 3). (**D**) Expression of stem cell markers was examined by qPCR in control HeLa‑vector cells and HeLa‑TBX1 cells (*n* = 6). For (**A-D**), the results are presented as mean ± s.e.m.; *P ≤ 0.05, **P ≤ 0.01, ***P ≤ 0.001; NS, not significant; unpaired two-tailed Student’s *t*-test.

To elucidate whether TBX1 can also influence EMT-associated stem cell properties, we compared CD44^+^CD24^−^ stem cell populations in HeLa cells stably transfected with a control vector or TBX1. CD44 is upregulated during EMT and in stem cells, and promotes cancer progression and metastasis^27^. As measured by flow cytometry, stable expression of TBX1 significantly diminished the percentage of CD44^+^CD24^−^ stem cells (Fig. 2A). Proliferation was also reduced in TBX1-overexpressing cells (Fig. 2B; Supplementary data, Fig. S3A-B). Furthermore, TBX1-overexpressing clones showed reduced sphere-forming capacity (Fig. 2C), associated with reduced expression of stem cell factors, *BMI1* and *SOX2* (Fig. 2D). In addition, TBX1 induced the expression of differentiation marker *KRT17* (Supplementary data, Fig. S3C). These findings suggest that TBX1 suppresses stem cell properties and induces epithelial differentiation.

### TBX1 is a regulator of miR-200

**Figure 3:**
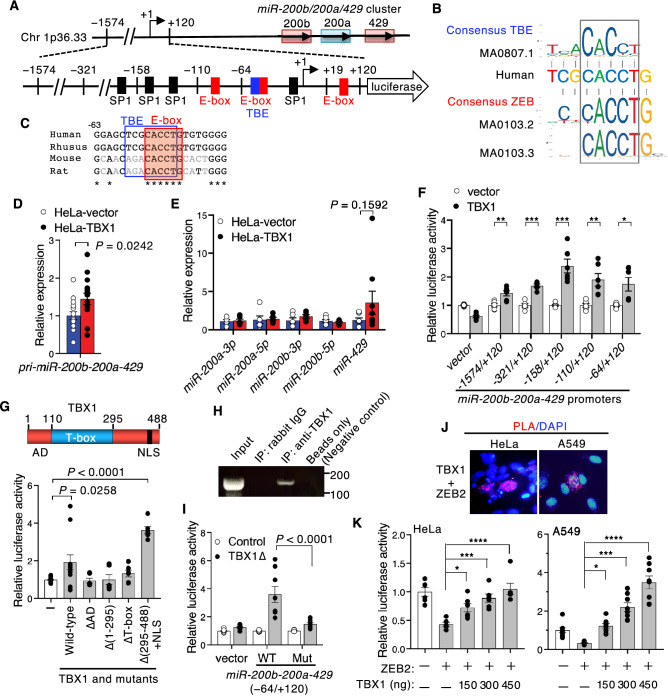
TBX1 induces *pri-miR-200b*-*200a*-*429.* (**A**) Schematic of the upstream promoter region of the human *miR-200b/200a/429* cluster on chromosome 1p36.33. The luciferase construct of the *miR-200b-200a-429* promoter with the location of the predicted TBE (blue box), as well as ZEB-binding E-boxes (red boxes)^15^ and SP1-binding sites (black boxes)^49^, are shown. (**B**) Sequence of TBE/E-box from the *miR-200b-200a-429* promoter and the JASPAR sequence logos of TBE and E-box motifs. The overlapping core sequence, CACCT, is boxed. (**C**) Nucleotide conservation of the *cis*-overlapping motifs of TBE and E-box. Asterisks indicate evolutionarily conserved nucleotides. (**D**) qPCR analysis of *pri-miR-200b-200a-429* expression in control HeLa‑vector cells and HeLa‑TBX1 cells (*n* = 15, *P ≤ 0.05; unpaired two-tailed Student’s *t*-test). (**E**) qPCR analysis of miRNAs in HeLa‑vector cells and HeLa‑TBX1 cells. Expression was normalized to *U6* and compared with that of the control (*n* = 6; Student’s *t*-test). (**F**) Relative luciferase activity of *miR-200b-200a-429* promoter reporters in HeLa cells (*n* = 9; Student’s *t*-test). (**G**) Top, schematic representation of TBX1 functional domains. Bottom, relative luciferase activity of the *miR-200b-200a-429* promoter constructs (− 321/ + 120) transiently transfected with various TBX1 deletion mutants (*n* = 6, *P ≤ 0.05, ***P ≤ 0.001; one-way ANOVA). AD, activation domain; NLS, nuclear localization signal. (**H**) Binding of TBX1 to the *miR-200b-200a-429* promoter (− 110/ + 19) detected by a ChIP assay using HeLa cells. (**I**) Relative luciferase activity of *miR-200b-200a-429* promoter constructs (− 64/ + 120) for wild-type (WT) or mutant (Mut) TBE transiently transfected with TBX1[∆(295–488) + NLS] in HeLa cells. Fold activity was measured in the absence of TBX1 and normalized to 1.0 (*n* = 8, two-way ANOVA). (**J**) In situ PLA detection of the TBX1 and ZEB2 colocalization. Constructs encoding TBX1 and ZEB2 were transfected into HeLa or A549 cells. PLA showed a very close proximity between TBX1 and ZEB2 at the nucleus, indicated by fluorescent spots (red). Nuclei were counterstained with DAPI (see also Supplementary data, Fig. S4). (**K**) Relative luciferase activity of HeLa or A549 cells transiently co-transfected with the *miR-200b-200a-429* promoter construct (− 321/ + 120), ZEB2 (150 ng), and the indicated amount of TBX1 expression vector (*n* = 6, *P ≤ 0.05, **P ≤ 0.01, ***P ≤ 0.001, ****P ≤ 0.0001; NS, not significant; one-way ANOVA).

The stem cell factors *BMI1* and *SOX2* are target genes of miR-200^24^. Given the association of TBX1 with epithelial differentiation and stem cell properties following reduced expression of miR-200 target genes (*ZEB2, BMI1,* and *SOX2*) we surmised that TBX1 may induce the expression of miR-200, which otherwise triggers E-cadherin/CDH1 by downregulating *ZEB2*^26^. To address this possibility, we focused on the EMT-suppressor *miR-200b-200a-429* as a potential novel target gene of TBX1. miR-200b, miR-200a, and miR-429 are clustered on human chromosome 1p36.33 (Fig. 3A). The *miR-200b-200a-429* promoter contains *cis*-regulatory motifs of the ZEB-binding E-box^15^. Using JASPAR, we identified a putative TBX1-binding element (TBE) within − 110/ + 120 of the *miR-200b-200a-429* transcription start site (Fig. 3A,B). Overlapping *cis*-regulatory E-box and TBE motifs were conserved among primates (Fig. 3C). Expression of *pri-miR-200b*-*200a*-*429* was significantly elevated in TBX1-overexpressing cells relative to control cells (Fig. 3D). Among the miR-200 family members, miR-429 was the most elevated by TBX1 overexpression, although the increase was not statistical significance (Fig. 3E). Because the *miR-200b-200a-429* promoter contains a TBE motif and TBX1 can induce the expression of *pri-miR-200b-200a-429* (Fig. 3A–D), we speculated that the observed effects were at least partly due to the activation of the *miR-200b-200a-429* promoter. As expected, TBX1 mildly activated miR-200 promoter constructs (Fig. 3F). We also searched for TBX1 functional domains for *miR-200b-200a-429* promoter activation, using TBX1 deletion mutants. Mutants with deletions of the activation domain (ΔAD) and T-box DNA-binding domain (ΔT-box) failed to activate the *miR-200b-200a-429* transactivation function (Fig. 3G), suggesting that the activation and DNA-binding domains are essential for *miR-200b-200a-429* promoter activation. Interestingly, TBX1[∆(295–488) + NLS] (hereafter TBX1∆) activated the *miR-200b-200a-429* promoter effectively (Fig. 3G). Chromatin immunoprecipitation (ChIP) showed that TBX1 directly bound to the − 110/ + 19 region of the *miR200b-200a-429* promoter (Fig. 3H). Mutations in TBE inhibited the stimulation of the *miR-200b-200a-429* promoter by TBX1Δ (Fig. 3I). In situ proximity ligation assay (PLA) showed a very close proximity between TBX1 and ZEB2 at the nucleus in HeLa cells and A549 human lung epithelial cells (Fig. 3J and Supplementary data, Fig. S4). Consistent with the previous reports^15^, overexpression of ZEB2 suppressed the activity of the *miR-200b-200a-429* promoter in HeLa and A549 cells (Fig. 3K). Inhibition of the *miR-200b-200a-429* promoter by ZEB2 was rescued by TBX1 in a dose-dependent manner in these cells (Fig. 3K), but not in MCF7 breast cancer cells (Supplementary data, Fig. S5). These findings demonstrate that TBX1 specifically rescues ZEB2-dependent inhibition of the *miR-200b-200a-429* promoter.

### TBX1 is a regulator of miR-203

**Figure 4:**
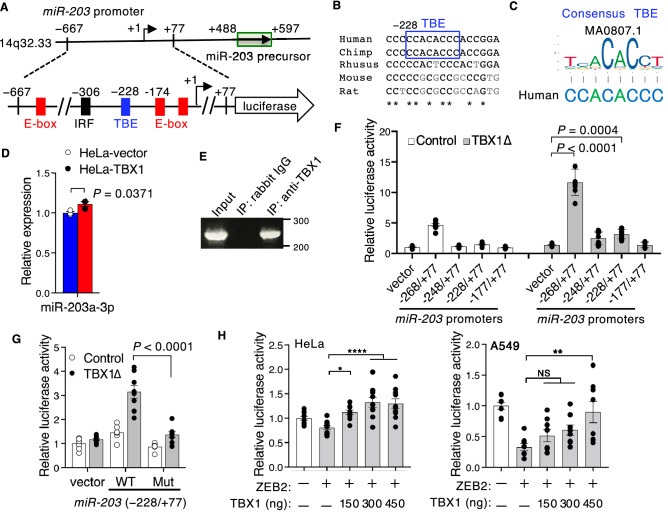
TBX1 induces miR-203*.* (**A**) Schematic of the upstream promoter region of the hsa-mir-203 stem-loop on chromosome 14q32.33 and the luciferase construct of the *miR-203* promoter with the location of the predicted TBE (blue box), as well as ZEB1-binding E-boxes (red boxes)^24^ and an IRF1-binding site (black box)^50^. (**B**) Nucleotide conservation of the *miR-203* promoter, including TBE. Asterisks indicate evolutionarily conserved nucleotides in all five species. (**C**) Sequence of TBE from the human *miR-203* promoter and the JASPAR sequence logos of TBE. (**D**) Expression of miR-203a-3p examined using qPCR in control HeLa‑vector cells and HeLa‑TBX1 cells. The graph shows the relative miRNA quantities (*n* = 3, *P ≤ 0.05; unpaired two-tailed Student’s *t*-test). (**E**) Binding of TBX1 to the *miR-203* promoter (− 298/ − 66) as detected by ChIP assay using HeLa cells. Chromatin was immunoprecipitated with normal rabbit IgG or anti-TBX1 antibodies. Input control is also shown. (**F**) Relative luciferase activity of *miR-203* promoter reporters in HeLa cells (*n* = 9, *P ≤ 0.05, ***P ≤ 0.001, ****P ≤ 0.0001; one-way ANOVA). (**G**) Relative luciferase activity of *miR-203* promoter constructs (− 228/ + 77) for wild-type (WT) or mutant (Mut) TBE transiently transfected with TBX1Δ (*n* = 8, two-way ANOVA). (**H**) Relative luciferase activity of HeLa or A549 cells transiently co-transfected with the *miR-203* promoter construct (− 667/ + 77), ZEB2 (150 ng), and the indicated amount of TBX1 expression vector (*P ≤ 0.05, **P ≤ 0.01, ****P ≤ 0.0001; NS, not significant; one-way ANOVA).

miR-203 is a stemness-inhibiting miRNA that is highly expressed in the epithelium^23^ and is downregulated in the stem cell population^28,29^. The polycomb repressor BMI1, whose expression is repressed in TBX1-overexpressing cells (Fig. 2D), is a common target of miR-200 and miR-203^24^. Therefore, we surmised that *miR-203* may be an additional downstream target of TBX1 along with *miR-200b-200a-429*. The *miR-203* promoter contains E-boxes^24^ and a putative TBE motif, which is conserved among primates (Fig. 4A–C). As expected, *miR-203a-3p* expression was mildly elevated in HeLa cells transfected with TBX1 (Fig. 4D). ChIP analysis showed that TBX1 directly bound within the − 298/ − 66 region of the human *miR-203* promoter (Fig. 4E). TBX1∆ activated the *miR-203* promoter constructs, which include a TBE motif (Fig. 4F). Mutations in TBE inhibited the stimulation of *miR-203* promoter (− 228/ + 77) by TBX1∆ (Fig. 4G). Consistent with previous reports^24^, overexpression of ZEB2 mildly suppressed *miR-203* promoter activity (Fig. 4H). TBX1 rescued ZEB2-dependent inhibition of the *miR-203* promoter in a dose-dependent manner (Fig. 4H). We also examined the effects of ZEB2 and TBX1 on the activities of artificial promoter, p4×RE-Luc, which contained four tandem copies of overlapping *cis*-regulatory E-box and TBE motifs (Supplementary data, Fig. S6). Overexpression of ZEB2 suppressed the p4×RE-Luc activity (Supplementary data, Fig. S6C). TBX1 hardly affected the ZEB2-dependent inhibition of p4×RE-Luc (Supplementary data, Fig. S6C)*.* These findings suggest that TBX1 reverses the inhibitory activity of ZEB2 through TBE, but the TBE sequence alone is not sufficient to rescue ZEB2 activity.

### TBX1 links the expression of epithelial miRNAs and their target genes in vivo

**Figure 5:**
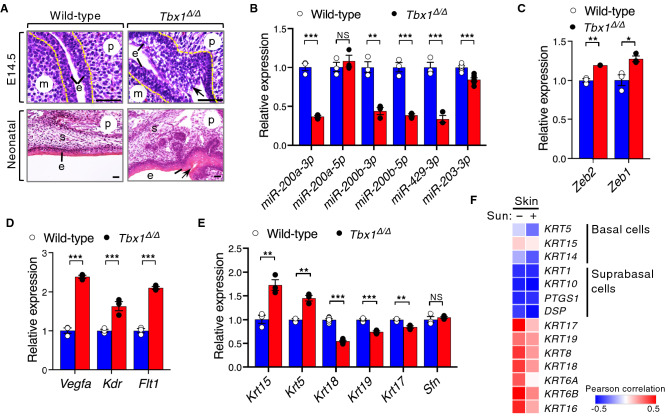
TBX1-mediated miRNA regulation engages epithelial differentiation in vivo. (**A**) Hematoxylin and eosin staining of the palate from wild-type and *Tbx1*^*Δ/Δ*^ embryos at the indicated age. Irregularities in the shape and multiple layers of the epithelium are evident in the *Tbx1*^*Δ/Δ*^ embryos (arrows). The broken yellow line traces the basal lamina. m, mandible; p, palatal shelf/palate; e, epithelium; s, stroma. Scale bars, 25 µm. (**B**) qPCR analysis of miRNAs in wild-type and *Tbx1*^*Δ/Δ*^ palatal shelves (*n* = 8 per genotype) at E13.5. Expression was normalized to that of *U6* and compared with that in wild-type littermates (*n* = 6). (**C**) qPCR analysis of *Zeb2* and *Zeb1* expression in wild-type and *Tbx1*^*Δ/Δ*^ palatal shelves (*n* = 8 per genotype) at E13.5. Expression was normalized to that of *Actb* and compared with that in wild-type littermates (*n* = 6). (**D**) qPCR analysis of *Vegfa*, *Kdr,* and *Flt1* expression in wild-type and *Tbx1*^*Δ/Δ*^ palatal shelves (*n* = 8 per genotype) at E13.5 (*n* = 6). (**E**) qPCR analysis of the expression of epithelial markers in wild type and *Tbx1*^*Δ/Δ*^ palatal shelves (*n* = 8 per genotype) at E13.5 (*n* = 3). (**F**) Heat map representing the pairwise correlation coefficients of the expression of *TBX1* and epithelial differentiation markers^51^ in the skin: sun-exposed (+ ; lower leg, *n* = 701) and unexposed (− ; suprapubic, *n* = 604). Pearson correlation coefficients (*r*) were calculated according to the color scale. Dark red denotes high positive correlation (*r* = 0.5), dark blue denotes high negative correlation (*r* =  − 0.5), and white denotes a lack of correlation (*r* = 0). The statistical source data are provided in Supplementary data, Figs S9–S11. For (**B-E**), the results are presented as mean ± s.e.m.; *P ≤ 0.05, **P ≤ 0.01, ***P ≤ 0.001; NS, not significant; unpaired two-tailed Student’s *t*-test.

To provide physiological evidence for the regulation of epithelial miRNAs and proliferation/differentiation balance by TBX1, we validated our findings in the palatal development of *Tbx1*^*Δ/Δ*^ mice, which exhibit cleft palate as the consequence of abnormal fusion between the epithelia covering the palatal shelves and the mandible^4^. As previously reported^4^, *Tbx1*^*Δ/Δ*^ embryos exhibit intraoral epithelial adhesions (Fig. 5A). The palatal epithelium of *Tbx1*^*Δ/Δ*^ neonates showed basal cell hyperplasia and multiple layers of the palatal epithelium (Fig. 5A). *Tbx1* ablation significantly reduced the expression of miR-200a-3p, miR-200b-3p, miR-200b-5p, miR-429-3p, and miR-203-3p in palatal shelves compared to controls (Fig. 5B). Palatal shelves from *Tbx1*^*Δ/Δ*^ mice also showed significantly increased expression of *Zeb2* and *Zeb1* (Fig. 5C). These findings demonstrate that *Tbx1* is required for the proper expression of miR-200, miR-203, and their targets, *Zeb1* and *Zeb2*, during palatogenesis.

**Figure 6:**
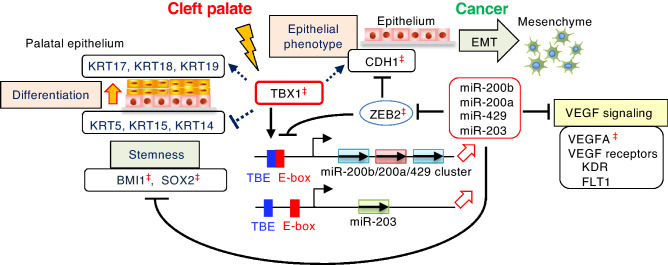
Proposed model of TBX1-mediated regulation of stemness and differentiation in epithelial cells through the miR-200–ZEB2 axis. ZEB2 and miR-200 reciprocally repress each other’s expression and are linked in a double-negative feedback loop^12,13,15^. TBX1 rescues ZEB2-dependent inhibition of the *miR-200b-200a-429* promoter and induces the expression of miR-200, which controls the expression of EMT- and VEGFA-related molecules. In addition, TBX1 induces miR-203 expression, which, together with miR-200, inhibits the expression of stem cell factors. In conclusion, TBX1 links the maintenance of stemness and epithelial differentiation, pathologically in epithelial cervical cancer, and physiologically in palatal development.^‡^, molecules associated with cleft palate in humans and/or mice.

The predicted target genes of miR-200 and miR-203 include human and mouse genes associated with syndromic cleft lip and/or palate (CL/P) (Supplementary data, Fig. S7). *VEGFA* and its receptor genes, *KDR* and *FLT1,* are known RNA targets of miR-200b-3p and miR-429-3p^20,21^. *Vegfa* is highly expressed in the midline of the developing palate, and *Vegfa* ablation causes cleft palate and craniofacial defects in mice^30^. These defects are reminiscent of those found in patients with DiGeorge syndrome^30^, suggesting that VEGF signaling may act in the genetic pathway of *TBX1*. Based on these findings, we speculated that TBX1 may indirectly affect the expression of *Vegfa* and its receptor genes, *Kdr* and *Flt1,* via miR-200 and miR-203 in palatal shelves. The predicted miR-200 and miR-203 binding sites in the 3′UTR of these genes were conserved throughout evolution (Supplementary data, Figs. S8 and S9). In the *Tbx1*^*Δ/Δ*^ palatal shelves, the expression of *Vegfa, Kdr,* and *Flt1* was significantly upregulated compared to that in the controls (Fig. 5D), suggesting that TBX1 regulates the genes that are involved in VEGF signaling. Moreover, loss of *Tbx1* correlates with an increase in the expression of epithelial basal cell markers (*Krt15* and *Krt5*) with poor epithelial differentiation (based on *Krt18, Krt19, Krt17* expression), indicating that *Tbx1* is essential for oral epithelial differentiation (Fig. 5E). Consistent with the *Tbx1*^*Δ/Δ*^ palatal model, we noted that *TBX1* expression was positively correlated with the expression levels of basal cell markers and negatively correlated with the expression levels of differentiation markers in skin cells (Fig. 5F, Supplementary data, Figs S10-12). The spatio-temporal regulation of differentiation of palatal epithelium is essential in preventing abnormal adhesion of oral epithelial surfaces during palatogenesis^31^. *Tbx1*^*Δ/Δ*^ palatal shelves exhibit a hyperproliferative epidermis that fails to undergo differentiation leading to cleft palate^4^. Together, our findings indicate that TBX1 is associated with miRNAs involved in regulating EMT and stemness, both pathologically in epithelial cancer cells and physiologically in epithelial differentiation (Fig. 6).

## Discussion

The miR-200–ZEB feedback loop has been extensively studied in various tumors to understand the mechanisms regulating miR-200 expression, which controls EMT and stem cell properties in epithelial cancer cells^9,32,33^. In this study, we discovered that TBX1 acts as a stemness inhibitor by rescuing ZEB2-dependent transcriptional inhibition of the promoters of miR-200 and miR-203. Our finding that TBX1 induces *miR-200b-200a-429* expression further substantiates the notion that miR-200 target genes, *ZEB2*, *BMI1* and *SOX2,* are downstream of TBX1. TBX-binding TBE and ZEB-binding E-box motifs have an overlapping core sequence of CACCT; therefore, the T-box family of transcription factors may have a counter-regulatory effect on ZEB-target genes, similar to *miR-200b-200a-429*. Regulatory mechanisms of TBX1 and the expression of *ZEB1* and *ZEB2* may differ among tumor types, organs, or species; however, the complete molecular mechanism underlying this effect is unknown. TBX1∆ activates the promoters of *miR-200b-200a-429* and *miR-203* effectively compared to wild-type TBX1; however, the molecular mechanisms and functions of TBX1∆ remain unknown. Additional studies are needed to identify novel common target genes and regulatory mechanisms of TBX1 and ZEB proteins.

CL/P is one of the most frequent craniofacial congenital disorders in humans^34^. Understanding the mechanisms regulating the balance between epithelial proliferation and differentiation during palatogenesis may have implications for preventing CL/P. The hyperproliferative oral epithelium in *Tbx1*^*Δ/Δ*^ mice failed to undergo epithelial differentiation, which may result in pathogenic intraoral adhesions^4^, similar to *Irf6*-mutant mice^35^. We provide evidence that *Tbx1* has molecular interactions with miR-200, miR-203, and their target genes, *Zeb2* and *Zeb1,* in mouse palatogenesis. Interestingly, both *Tbx1*^*Δ/Δ*^ and *Zeb2*^+*/Δ*^*;Zeb1*^*Δ/Δ*^ mouse embryos exhibit unique CL/P phenotypes of varying severity, ranging from incomplete to complete cleft palate^4,18^. In humans, missense mutations in *ZEB2* induce Mowat–Wilson syndrome associated with CL/P (OMIM #235,730)^36,37^. In addition to *ZEB2* and *Zeb1*, predicted targets of miR-200 include disease genes related to CL/P in humans and mice. These results suggest that primary defects in regulators and target genes of miR-200 are significant contributors to CL/P. Genes that play essential roles in tumorigenesis are often involved in cell proliferation and differentiation during embryogenesis^38^. For individuals born with CL/P, an increased risk of breast cancer was observed^39^. Thus, the TBX1-mediated link between miR-200 and its target genes may not be a unique phenomenon in selected tumors; rather, it may be a regulatory system already active in embryonic development. *Tbx1* ablation decreased miR-200 and miR-203 in palatal shelves and was associated with increased expression of their target genes, *Vegfa, Kdr*, and *Flt1*. *Vegfa* ablation causes cardiovascular, craniofacial, thymic, and parathyroid defects in mice, reminiscent of the birth anomalies found in *Tbx1*^*Δ/Δ*^ mice^30^. *Tbx1* may downregulate VEGF signaling, at least in part, through miR-200 and miR-203. Since most anti-angiogenic strategies focus on inhibiting VEGF and its receptors^22^, controlling the miR-200–ZEB2–VEGF signaling axis by TBX1 may be an essential pathway for influencing tumor progression. The repressive influence of transiently-expressed *Tbx1* may be released temporally and spatially in order for the expression of miR-200 and miR-203 to regulate their target genes and execute epithelial differentiation during palatogenesis. Because our data were derived from ectoderm-derived epithelium in the developing palate, further validation of miR-200 in *Tbx1-*expressing endoderm-derived epithelia is needed to confirm our findings.

In conclusion, TBX1 directly or indirectly links the expression of miRNAs and EMT/stemness-related factors pathologically in epithelial cervical cancer and physiologically in palatal development. We propose that TBX1 induces epithelial differentiation and inhibits stemness by activating the expression of EMT/stemness-inhibiting miRNAs and consequently repressing the expression of their target genes. The finding that TBX1 regulates stem cell properties through association with miR-200–ZEB2 suggests possible strategies for developing personalized medicine for treating patients with cervical cancer by maintaining a differentiated epithelial cell phenotype. In addition, the miR-200–ZEB2 axis is involved in TBX1-mediated control of epithelial differentiation during palatogenesis, contributing to our understanding of CL/P etiology.

## Materials and methods

### Bioinformatic analysis

Survival analysis was performed by stratifying patients into high, medium, or low *TBX1* expression groups, and Kaplan–Meier plots were generated and analyzed using the UCSC Xena program (https://xenabrowser.net)^40^ The mRNA expression data of tumors, matched normal specimens, sun-exposed skin, and unexposed skin were obtained from GEPIA (http://gepia.cancer-pku.cn/detail.php)^41^using the GTEx (https://gtexportal.org/home) or TCGA (http://cancergenome.nih.gov) database. The JASPAR database (http://jaspar.genereg.net) of transcription factor binding site motifs^42^ was used to predict putative TBEs and ZEB consensus E-box motifs. The predicted target genes of miRNAs were identified using TargetScan 7.2 (http://www.targetscan.org). Correlations between mRNA–mRNA pairs of the gene set were analyzed by calculating the Pearson correlation coefficient.

### Plasmids

The pEBMulti-Hyg-TBX1 vector^4^ was generated from the EBNA1-based episomal pEBMulti-Hyg vector (Fujifilm Wako), which included a hygromycin-resistant gene. Expression vectors of Myc-tagged TBX1, its deletion mutants, and p4×RE-Luc were previously described^43,44^. To construct the ZEB2 plasmid, the cDNA sequence was amplified and cloned into pFlag-CMV3.1 (ThermoFisher Scientific). The *miR-200b-200a-429* promoter (− 1574/ + 120) and *miR-203* promoter (− 667/ + 77) were amplified from human genomic DNA; full-length DNA, and deletion mutants were then directionally cloned into the pGL2-Basic vector (Promega). Primer sequences used to construct the vectors are shown in Supplementary data, Table S1.

### Cell culture and stable transfection of cell lines

The cell lines HeLa (Tohoku University cell bank), A549 (RIKEN cell bank), and MCF7 (a kind gift from Dr. K. Ito) were cultured in DMEM (Nacalai Tesque) supplemented with 1% penicillin–streptomycin and 10% fetal bovine serum (FBS). Cells were transfected with pEBMulti-Hyg or pEBMulti-Hyg-Myc-TBX1 vectors using the Avalanche-Everyday transfection reagent (Integral) or FuGENE HD (Promega). The transfected cells were then selected using 300 μg/mL (HeLa) or 400 μg/mL (MCF7) hygromycin (Nacalai Tesque).

### Mouse strains

All animal care and experiments were conducted in accordance with the ARRIVE (Animal Research: Reporting of In Vivo Experiments) guidelines. Heterozygous mice (*Tbx1*^neo/+^) were mated with *More-Cre* mice (a kind gift from Dr. M. Tallquist), resulting in the heterozygous *Tbx1*-null allele (*Tbx1*^+/*Δ*^) and *Tbx1* homozygous-null *Tbx1*^*Δ/Δ*^ mice, as described previously^4,6^. Wild-type littermates were used as controls. All experimental animal procedures were reviewed and approved by the Institutional Animal Care and Use Committee of Tokyo Medical and Dental University (Permit Number 0126215C, February 24, 2016). All experiments and methods were performed in accordance with relevant guidelines and regulations.

### Migration and invasion assays

Assays were conducted in 24-well Transwell chambers (6.5 mm, 8.0 μm pore size; Corning) pre-coated without (for migration assays) or with (for invasion assays) 50 µL of Matrigel (BD Biosciences). For migration assays, 1.0 × 10^5^ cells in 100 μL of serum-free medium were placed in the upper compartment of the Transwells. The lower compartment was filled with 600 μL of the complete culture medium. After incubation at 37 °C for 24 h, the migrated cells that passed through the pores on each insert were counted. For the invasion assay, the cells that penetrated the Matrigel-coated membrane into the lower side were fixed with 3.7% formaldehyde in phosphate-buffered saline (PBS) and stained with 0.1% crystal violet. Invaded cells were imaged in three individual fields on each insert using a BX41 microscope (Olympus) and counted using Fiji (https://imagej.net/software/fiji).

### Soft agar colony formation assay

Anchorage-independent growth of HeLa-vector and HeLa-TBX1 stably transfected cells was estimated using a soft agar colony formation assay, as described previously^45^. Cells were suspended in 1.5 mL of DMEM containing 10% FBS, 300 μg/mL hygromycin, and 0.3% agarose and plated at 7500 cells/well in triplicate over a 1.5 mL 0.6% agarose base in 6-well plates. Fresh medium (300 μL) was added to the plate for 3 weeks. The colonies in the plate were imaged using a BX41 microscope and analyzed using Fiji.

### Quantitative real-time PCR (qPCR) analysis

Palatal shelves were dissected from wild-type and *Tbx1*^*Δ/Δ*^ mice (*n* = 8 per genotype) on embryonic day (E)13.5. Cultured cells and secondary palatal shelves were processed for total RNA extraction using TRIzol (ThermoFisher) and the RNeasy Mini Kit (Qiagen). mRNA analysis was carried out using the 1st strand cDNA synthesis kit for RT-PCR (AMV) (Sigma-Aldrich) and PowerUP SYBR Green PCR Master Mix (ThermoFisher). mRNA expression was normalized to that of *TBP* or *Actb*. miRNA analysis was performed as previously described^46^. Briefly, cDNA was synthesized from total RNA, and miRNAs were amplified using an All-in-One miRNA qRT-PCR Detection Kit (GeneCopoeia) with stem-loop primers specifically designed to analyze mature miRNAs. The expression of miRNAs was normalized to the endogenous control *U6* (GeneCopoeia). Amplification and detection of mRNAs and miRNAs were performed using the StepOnePlus Real-Time PCR System (ThermoFisher), and the relative quantity was calculated using the 2^ − ΔΔCt method^47^. Experiments were performed in at least three replicates for each sample and each gene. The primer sequences used for qPCR are shown in Supplementary data, Table S2.

### Western blotting

HeLa cells stably transfected with the indicated plasmids were lysed using RIPA lysis buffer (50 mM Tris–HCl [pH 7.5], 150 mM NaCl, 1 mM EDTA, 1% Triton X-100 supplemented with 1 × cOmplete protease inhibitor cocktail (Sigma-Aldrich). The cell lysates were electrophoresed via SDS–PAGE, transferred onto PVDF membranes (Amersham), blocked with 5% non-fat dried milk (BD Biosciences), and incubated with appropriate primary and HRP-conjugated secondary antibodies (Bio-Rad Laboratories). Immunoblots were visualized using an ECL detection system (Santa Cruz Biotechnology). The Western blots were imaged using ChemiDoc MP (Bio-Rad). The following primary antibodies were used: rabbit anti-Myc (A14; Santa Cruz, 1:1,000), rabbit anti-E-cadherin (24E10; Cell Signaling Technology, 1:1,000), anti-vimentin (D21H3; Cell Signaling, 1:1,000), anti-N-cadherin (#32; BD Biosciences, 1:1,000), anti-β-catenin (6B3; Cell Signaling, 1:1,000), anti-β-tubulin (9F3; Cell Signaling, 1:1,000), and anti-DSG1 (H-290; Santa Cruz, 1:1,000). The full-length blots are shown in Supplementary data, Fig. S13.

### Flow cytometric analysis

Cells were collected with 0.05% trypsin–EDTA solution, washed, and resuspended at 5.0 × 10^6^/mL in PBS with 2% FBS (staining medium) containing PE-conjugated anti-CD24 (ML5, BD Biosciences) and FITC-conjugated anti-CD44 (G44-26, BD Biosciences) antibodies. Cells were incubated at 4 °C for 60 min, washed with PBS, resuspended in staining medium, and analyzed using FACSLyric (BD Biosciences). Side scatter and forward scatter profiles were used to eliminate debris and cell doublets. Standard compensation was performed using single-stained cells incubated with a labeled isotype antibody. A total of 10,000 viable cells were counted.

### Cell proliferation assays

For the vital carboxyfluorescein N-hydroxysuccinimidyl ester (CFSE) dye dilution assay, the CellTrace CFSE cell proliferation kit (ThermoFisher) was used according to the manufacturer’s instructions. Briefly, stable cell lines were collected with 0.05% trypsin–EDTA solution, washed with PBS, and resuspended in PBS at a final density of 1.0 × 10^6^/mL. Next, the cells were mixed with the CFSE fluorescent dye in PBS (final concentration 5 uM) and incubated for 20 min at 37 °C. Cells were then washed with serum-free medium, seeded in 10-cm dishes at 20% confluency, and cultured in DMEM containing 10% FBS. CFSE dilution resulting from cell division was evaluated using FACSLyric. Side scatter and forward scatter profiles were used to eliminate debris and cell doublets. For cell cycle analysis, cells were analyzed using the Muse Cell Cycle Kit (Merck). A total of 10,000 viable cells were counted. Each experiment was repeated three times using independent samples.

### Sphere-formation assay

To test sphere-forming capacity, cells were resuspended in DMEM containing 0.5% methylcellulose #1500 (Nacalai), 10% FBS, 300 μg/mL hygromycin, and 1% penicillin–streptomycin. 10,000 single cells were seeded into individual wells of 6-well clear flat-bottom ultra-low attachment plates (Coster, #3471). Colonies with a diameter greater than 80 µm were counted after 12 days.

### Reporter assays

HeLa, A549, and MCF7 cells were plated in 24-well plates and co-transfected with 150 ng of firefly luciferase reporter and expression plasmids using TransFectin lipid reagent (Bio-Rad Laboratories) or TransIT-2020 Transfection Reagent (Mirus Bio) along with 50 ng of a pRSV-β-galactosidase expression plasmid to monitor transfection efficiency. After 48 h of incubation, cells were lysed in passive lysis buffer (Promega), and luciferase activity was measured using the Luciferase Reporter Assay System (Promega). All data are expressed as mean ± standard error of the mean (s.e.m.) from at least six separate experiments.

### Chromatin immunoprecipitation (ChIP) assay

The ChIP assay was performed as previously described^48^, with minor modifications. After cells were formaldehyde cross-linked and lysed, sheared chromatin was immunoprecipitated with anti-TBX1 antibody (34–9800, ThermoFisher), and DNA was isolated and analyzed by PCR. The gels were imaged using BioDoc-It System (analytik jena US). The primer sequences are listed in Supplementary data, Table S3. The full-length gel is shown in Supplementary data, Fig. S14.

### Proximity ligation assay (PLA)

PLA was performed with the Duolink In Situ Detection Reagents-Red kit (Sigma-Aldrich) following the manufacturer’s instructions. Antibodies used for PLA were rabbit anti-Myc (A14; Santa Cruz) and mouse anti-Flag (M2; Sigma-Aldrich). Nuclei were counterstained with DAPI. Images were acquired by microscopy (BX-61; Olympus) and processed with Fiji.

### Statistical analysis

The log-rank test was used to compare the Kaplan–Meier survival curves. Correlation of mRNA-mRNA pairs of the gene set in cell lines or human tumors was analyzed by calculating the Pearson correlation coefficient. The experiments were repeated at least three times independently. Data were analyzed using PRISM 9.0 software (GraphPad) and were presented as mean ± s.e.m. for *n* experiments. Unpaired two-tailed Student’s *t*-tests were applied to compare two groups of independent samples. A one-way ANOVA with Dunnett’s post hoc test was used to analyze differences among three or more groups. Statistical significance is presented as follows: *P ≤ 0.05, **P ≤ 0.01, ***P ≤ 0.001 and ****P ≤ 0.0001.

## Supplementary Information

Supplementary Information.

## Data Availability

The Affymetrix expression data from GTEx analysed during the current study are available in the GEO repository, GSE45878. Other datasets generated during and/or analysed during the current study are included in this published article (and its Supplementary Information files) or available from the corresponding author on reasonable request.

## References

[1] Yagi H (2003). Role of TBX1 in human del22q11.2 syndrome. Lancet (London, England).

[2] Ogata T (2014). TBX1 mutation identified by exome sequencing in a Japanese family with 22q11.2 deletion syndrome-like craniofacial features and hypocalcemia. PLoS One.

[3] Zweier C, Sticht H, Aydin-Yaylagül I, Campbell CE, Rauch A (2007). Human TBX1 missense mutations cause gain of function resulting in the same phenotype as 22q11.2 deletions. Am. J. Hum. Genet..

[4] Funato N, Nakamura M, Richardson JA, Srivastava D, Yanagisawa H (2012). Tbx1 regulates oral epithelial adhesion and palatal development. Hum. Mol. Genet..

[5] Funato N, Nakamura M, Richardson JA, Srivastava D, Yanagisawa H (2015). Loss of Tbx1 induces bone phenotypes similar to cleidocranial dysplasia. Hum. Mol. Genet..

[6] Hu T (2004). Tbx1 regulates fibroblast growth factors in the anterior heart field through a reinforcing autoregulatory loop involving forkhead transcription factors. Development.

[7] Lindsay EA (2001). Chromosomal microdeletions: dissecting del22q11 syndrome. Nat. Rev. Genet..

[8] Trempus CS (2011). A novel role for the T-box transcription factor Tbx1 as a negative regulator of tumor cell growth in mice. Mol. Carcinog.

[9] Nieto MA, Huang RY-J, Jackson RA, Thiery JP (2016). EMT: 2016. Cell.

[10] Thiery JP, Acloque H, Huang RYJ, Nieto MA (2009). Epithelial-mesenchymal transitions in development and disease. Cell.

[11] Aban CE (2021). Downregulation of E-cadherin in pluripotent stem cells triggers partial EMT. Sci. Rep..

[12] Burk U (2008). A reciprocal repression between ZEB1 and members of the miR-200 family promotes EMT and invasion in cancer cells. EMBO Rep..

[13] Park SM, Gaur AB, Lengyel E, Peter ME (2008). The miR-200 family determines the epithelial phenotype of cancer cells by targeting the E-cadherin repressors ZEB1 and ZEB2. Genes Dev..

[14] Gregory PA (2008). The miR-200 family and miR-205 regulate epithelial to mesenchymal transition by targeting ZEB1 and SIP1. Nat. Cell Biol..

[15] Bracken CP (2008). A double-negative feedback loop between ZEB1-SIP1 and the microRNA-200 family regulates epithelial-mesenchymal transition. Cancer Res..

[16] Christoffersen NR, Silahtaroglu A, Orom UA, Kauppinen S, Lund AH (2007). miR-200b mediates post-transcriptional repression of ZFHX1B. RNA.

[17] Takagi T, Moribe H, Kondoh H, Higashi Y (1998). DeltaEF1, a zinc finger and homeodomain transcription factor, is required for skeleton patterning in multiple lineages. Development.

[18] Miyoshi T (2006). Complementary expression pattern of Zfhx1 genes Sip1 and deltaEF1 in the mouse embryo and their genetic interaction revealed by compound mutants. Dev. Dyn..

[19] Shin JO (2012). MiR-200b regulates cell migration via Zeb family during mouse palate development. Histochem. Cell Biol..

[20] McArthur K, Feng B, Wu Y, Chen S, Chakrabarti S (2011). MicroRNA-200b regulates vascular endothelial growth factor-mediated alterations in diabetic retinopathy. Diabetes.

[21] Choi YC, Yoon S, Jeong Y, Yoon J, Baek K (2011). Regulation of vascular endothelial growth factor signaling by miR-200b. Mol. Cells.

[22] Ellis LM, Hicklin DJ (2008). VEGF-targeted therapy: mechanisms of anti-tumour activity. Nat. Rev. Cancer.

[23] Yi R, Poy MN, Stoffel M, Fuchs E (2008). A skin microRNA promotes differentiation by repressing ‘stemness’. Nature.

[24] Wellner U (2009). The EMT-activator ZEB1 promotes tumorigenicity by repressing stemness-inhibiting microRNAs. Nat. Cell Biol..

[25] Burk RD (2017). Integrated genomic and molecular characterization of cervical cancer. Nature.

[26] Comijn J (2001). The two-handed E box binding zinc finger protein SIP1 downregulates E-cadherin and induces invasion. Mol. Cell.

[27] Al-Hajj M, Wicha MS, Benito-Hernandez A, Morrison SJ, Clarke MF (2003). Prospective identification of tumorigenic breast cancer cells. Proc. Natl. Acad. Sci. U. S. A..

[28] Chang CJ (2011). P53 regulates epithelial-mesenchymal transition and stem cell properties through modulating miRNAs. Nat. Cell Biol..

[29] Shimono Y (2009). Downregulation of miRNA-200c Links breast cancer stem cells with normal stem cells. Cell.

[30] Stalmans I (2003). VEGF: a modifier of the del22q11 (DiGeorge) syndrome?. Nat. Med..

[31] Richardson RJ, Dixon J, Jiang R, Dixon MJ (2009). Integration of IRF6 and Jagged2 signalling is essential for controlling palatal adhesion and fusion competence. Hum. Mol. Genet..

[32] Brabletz S, Brabletz T (2010). The ZEB/miR-200 feedback loop–a motor of cellular plasticity in development and cancer?. EMBO Rep..

[33] Mani SA (2008). The epithelial-mesenchymal transition generates cells with properties of stem cells. Cell.

[34] Tolarova MM, Cervenka J (1998). Classification and birth prevalence of orofacial clefts. Am. J. Med. Genet..

[35] Richardson RJ (2006). Irf6 is a key determinant of the keratinocyte proliferation-differentiation switch. Nat. Genet..

[36] Cacheux V (2001). Loss-of-function mutations in SIP1 Smad interacting protein 1 result in a syndromic Hirschsprung disease. Hum. Mol. Genet..

[37] Wilson M (2003). Further delineation of the phenotype associated with heterozygous mutations in ZFHX1B. Am. J. Med. Genet. A.

[38] Monk M, Holding C (2001). Human embryonic genes re-expressed in cancer cells. Oncogene.

[39] Bille C (2005). Cancer risk in persons with oral cleft - A population-based study of 8,093 cases. Am. J. Epidemiol..

[40] Goldman MJ (2020). Visualizing and interpreting cancer genomics data via the Xena platform. Nat. Biotechnol..

[41] Tang Z (2017). GEPIA: A web server for cancer and normal gene expression profiling and interactive analyses. Nucleic Acids Res..

[42] Fornes O (2020). JASPAR 2020: update of the open-access database of transcription factor binding profiles. Nucleic Acids Res..

[43] Funato N, Srivastava D, Shibata S, Yanagisawa H (2020). TBX1 regulates chondrocyte maturation in the spheno-occipital synchondrosis. J. Dent. Res..

[44] Weintraub H, Davis R, Lockshon D, Lassar A (1990). MyoD binds cooperatively to two sites in a target enhancer sequence: Occupancy of two sites is required for activation. Proc. Natl. Acad. Sci. U. S. A..

[45] Cifone MA, Fidler IJ (1980). Correlation of patterns of anchorage-independent growth with in vivo behavior of cells from a murine fibrosarcoma. Proc. Natl. Acad. Sci. U. S. A..

[46] Funato N (2020). The transcription factor HAND1 is involved in cortical bone mass through the regulation of collagen expression. Int. J. Mol. Sci..

[47] Schmittgen TD (2000). Quantitative reverse transcription-polymerase chain reaction to study mRNA decay: comparison of endpoint and real-time methods. Anal. Biochem..

[48] Shang Y, Hu X, DiRenzo J, Lazar MA, Brown M (2000). Cofactor dynamics and sufficiency in estrogen receptor-regulated transcription. Cell.

[49] Kolesnikoff N (2014). Specificity protein 1 (Sp1) maintains basal epithelial expression of the mir-200 family: Implications for epithelial-mesenchymal transition. J. Biol. Chem..

[50] Mao L, Zhang Y, Mo W, Yu Y, Lu H (2015). BANF1 is downregulated by IRF1-regulated MicroRNA-203 in cervical cancer. PLoS ONE.

[51] Bragulla HH, Homberger DG (2009). Structure and functions of keratin proteins in simple, stratified, keratinized and cornified epithelia. J. Anat..

