# Supplementary material for: TBX1 targets the miR-200–ZEB2 axis to induce epithelial differentiation and inhibit stem cell properties
Source: Sci Rep. 2022 Nov 23;12:20188. doi: 10.1038/s41598-022-24604-9 (PMC9684448; doi:10.1038/s41598-022-24604-9)
Supplement: Supplementary file 1 — Supplementary Information. [file 41598_2022_24604_MOESM1_ESM.pdf]

TBX1 targets the miR-200–ZEB2 axis to induce epithelial differentiation and inhibit stem cell properties

Supplementary Materials

Supplementary Table S1. Primer sequences for plasmid construction.

Primer	Sequence
miR-200 promoter_−1574_FW_NheI	5′-ACCTGCTAGCCATTCCCGGACAGTCACAG-3′
miR-200 promoter_−321_FW_NheI	5′-GTGGGCTAGCAGAGGTGGAGAGGCGAGAGT-3′
miR-200 promoter_−158_FW_NheI	5′-ATCGGCTAGCCTGCGTCACCGTCACT-3′
miR-200 promoter_−110_FW_NheI	5′-GTGAGCTAGCCAGGGGACACACCTGTCG-3′
miR-200 promoter_−64_FW_NheI	5′-GTCGGCTAGCGAGCTCGCACCTGTGTGG-3′
miR-200 promoter_−64_Mutant_FW_NheI	5′-GTCGGCTAGCGAGCTCG<u>TTT</u>CTGTGTGG-3′
miR-200 promoter_+120_RV_BglII	5′-CAGGAGATCTCCTGGCACAGGAAGTCAGTT-3′
miR-200 promoter_+19_RV_BglII	5′-TGGAAGATCTGGGGCCTCGGGAGGGAAGAG-3′
miR-203 promoter_−667_FW_XhoI	5′-CCGCTCGAGATGGCTCCAGACTTGGGGCA-3′
miR-203 promoter_+77_RV_HindIII	5′-CCCAAGCTTCGACTGATCCTCCACGGC-3′
miR-203 promoter_−268_FW_XhoI	5′-CCGCTCGAGAAGTGAGAGGGGCTGGGGTGG-3′
miR-203 promoter_−248_FW_XhoI	5′-CCGCTCGAGGGGTGTGTCCAGCCCAGCCCC-3′
miR-203 promoter_−228_FW_XhoI	5′-CCGCTCGAGCCACACCCACCGGAGAGCTAG-3′
miR-203 promoter_−177_FW_XhoI	5′-CCGCTCGAGCCTCACCTGTTCCGGCCACCC-3′
miR-203 promoter_−228_Mutant_FW_XhoI	5′-CCGCTCGAGCCA<u>TGG</u>CCACCGGAGAGCTAG-3′
Zeb2_FW_ClaI	5′-AAAATCGATATGAAGCAGCCGATCATG-3′
Zeb2_RV_SalI	5′-AAAGTCGACTTATTCCATGCCATCTTCC-3′

Underlined letters indicate the mutation in the promoter sequence.

Supplementary Table S2. Primer sequences for qPCR.

Gene	Sense Sequence (5′-3′)	Antisense Sequence (5′-3′)
TBX1	GCGCTGTGGGACGAGTTCAATCAG	GCACAAAGTCCATGAGCAGCATGTAGTC
ZEB1	TTACACCTTTGCATACAGAACCC	TTTACGATTACACCCAGACTGC
ZEB2	CAAGAGGCGCAAACAAGCC	GGTTGGCAATACCGTCATCC
BMI1	CGTGTATTGTTCGTTACCTGGA	TTCAGTAGTGGTCTGGTCTTGT
SOX2	TACAGCATGTCCTACTCGCAG	GAGGAAGAGGTAACCACAGGG
ETV7	CTGCTGTGGGATTACGTGTATC	GTTCTTGTGATTTCCCCAGAGTC
KRT17	GCCGCATCCTCAACGAGAT	CGCGGTTCAGTTCCTCTGTC
TBP	CAGCAACTTCCTCAATTCCTTG	GCTGTTTAACTTCGCTTCCG
pri-miR-200b-200a-429	TCGAAACTCTCCCAGAGACG	AGACCTGCAAGGGTGAGCTT
Vegfa	GCTTCCTACAGCACAGCAGA	AATGCTTTCTCCGCTCTGAA
Kdr	CAAACCTCAATGTGTCTCTTTGC	AGAGTAAAGCCTATCTCGCTGT
Flt1	CACTGACATACCCAAACTTGTGC	GTCCCATGTTATTCTTTGCCCAT
Zeb2	ATGAGCTTCCTACCGCATATG	GGCCTGACATGTAGTCTTGTG
Zeb1	AGACCAGACAGTATTACCAGGAG	ACATTCGGATCATGGTTTTGC
Krt15	GTCAATCTCCAGGACAACGC	CAGATCGGGACTACAGCCAT
Krt5	CACAAACTCATTCTCAGCCG	CAGAGCTGAGGAACATGCAG
Krt18	GACTCTAAAGTCATCGGCGG	CTGGGGCCACTACTTCAAGA
Krt19	CTGAAGTCATCTGCAGCCAG	AGACCATCGAGGACTTGCG
Krt17	ACCATCCGCCAGTTTACCTC	CTACCCAGGCCACTAGCTGA
Sfn	TTGGCTGAACAGGCCGAAC	GTAAGCTACGGAAAGCAGGTTT
Tbx1	GCGCTGTGGGACGAGTTCAATCAG	GCACAAAGTCCATGAGCAGCATGTAGTC
Kdr	CAAACCTCAATGTGTCTCTTTGC	AGAGTAAAGCCTATCTCGCTGT
Flt1	CACTGACATACCCAAACTTGTGC	GTCCCATGTTATTCTTTGCCCAT
Actb	ATGGAGGGGAATACAGCCC	TTCTTTGCAGCTCCTTCGTT

Supplementary Table S3. Primer sequences for ChIP.

Primer	Sequence
miR-200 promoter_–110_FW	5′-CAGGGGACACACCTGTCG-3′
miR-200 promoter_+19_RV	5′-GGGGCCTCGGGAGGGAAGAG-3′
miR-203 promoter_–298_FW	5′-TCTCTTTACCCCTCCCCTTC-3′
miR-203 promoter_–66_RV	5′-CCGAACCGTCTCGTCTGG-3′

Supplementary Figure S1

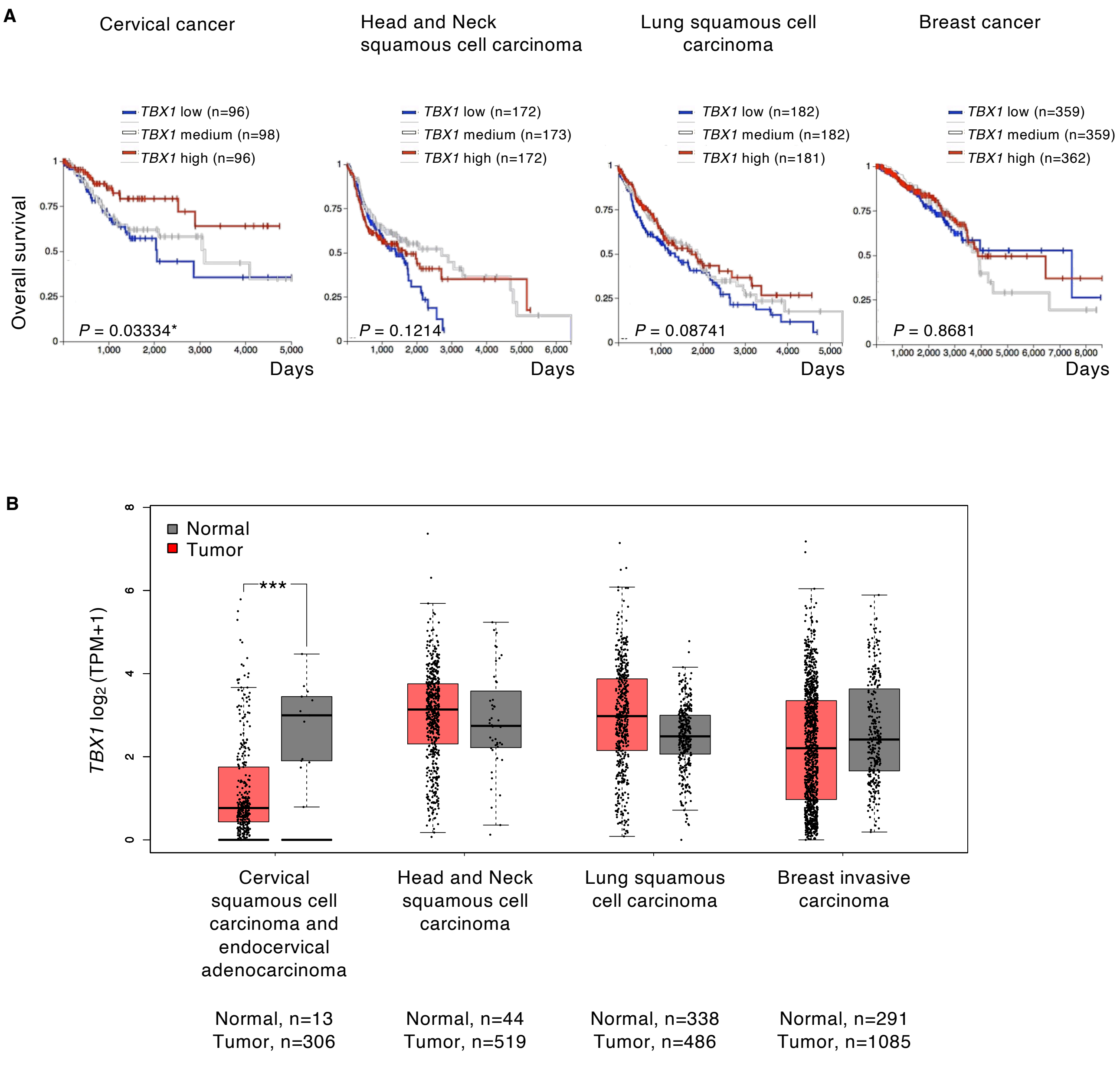

Supplementary Figure S1. Overall survival of patients with *TBX1*-expressing carcinomas.
(**A**) Overall survival of patients with primary head and neck cancer, lung squamous cell carcinoma, and breast cancer with high, medium, or low *TBX1* expression. Two-sided *P*-values were computed using a log-rank (Mantel-Cox) test.
(**B**) Box plots obtained from GEPIA using the GTEx database to compare the expression of *TBX1* in tumors and matched normal specimens. TPM, transcripts per million. ***$P \leq 0.001$; one-way ANOVA.

Supplementary Figure S2

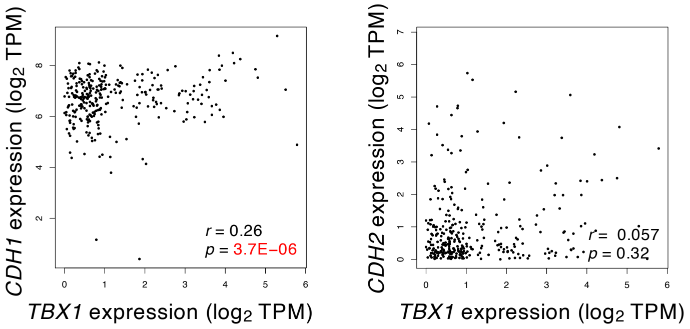

Supplementary Figure S2. Correlation of *TBX1* and *CDH1* or *CDH2* gene expression*.*
Positive correlation between *TBX1* and *CDH1*, but not *CDH2,* was observed in cervical cancer tissues analyzed by GEPIA using TCGA database. *r,* Pearson correlation coefficient; *p,* significance level.

Supplementary Figure S3

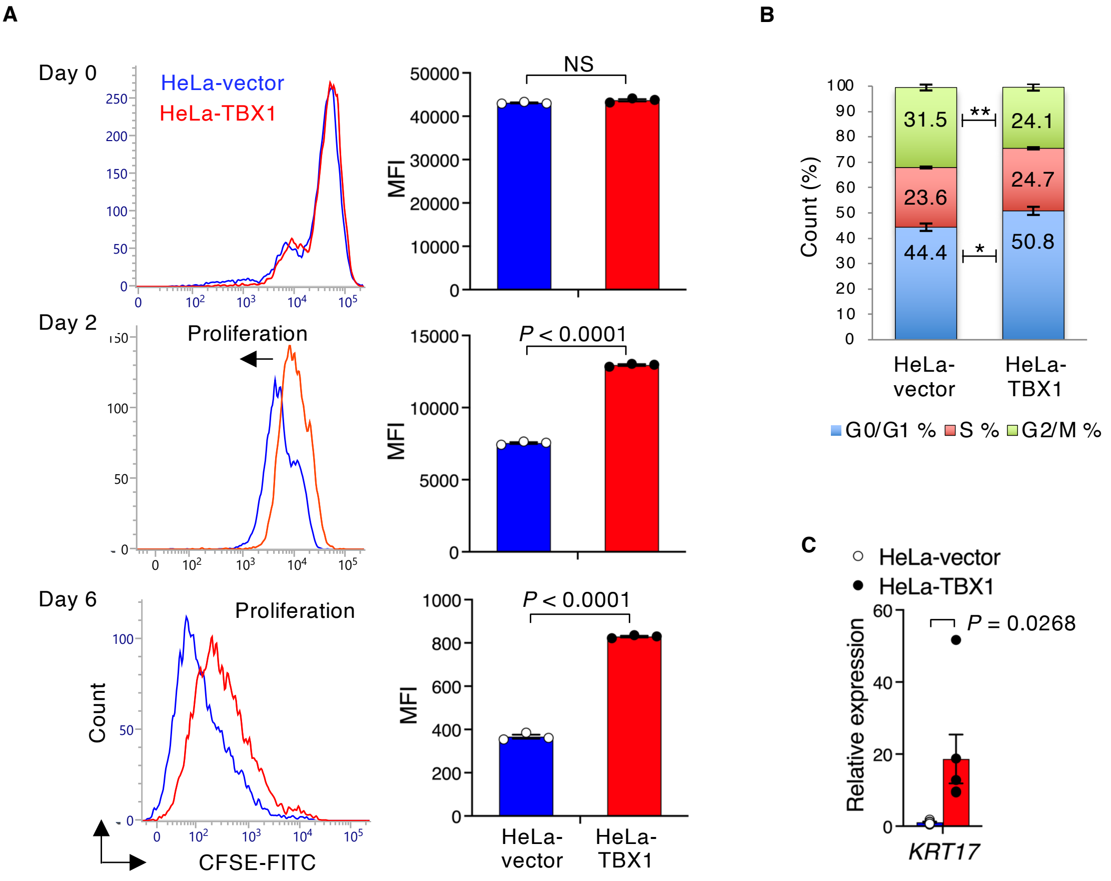

Supplementary Figure S3. TBX1 suppresses proliferation and induces expression of the epithelial differentiation marker *KRT17*.

(**A**) Flow cytometry analysis of empty vector and TBX1 stably transfected HeLa cells labeled with the CFSE fluorescent dye: representative day 0, day 2, and day 5 histograms (left) and means ±s.e.m. of quantified results from three experiments with replicate wells (right) are shown. NS, not significant.

(**B**) HeLa cells overexpressing TBX1 show reduced proliferation as determined by cell cycle analysis (n = 6). *$P \leq 0.05$, **$P \leq 0.01$.

(**C**) Expression of *KRT17* examined by qPCR in HeLa cells stably transfected with control pEBMulti plasmid (vector) or pEBMulti-TBX1 plasmid (TBX1) (n = 6).

For **A-C**, the results are presented as mean ± s.e.m.; unpaired two-tailed Student's t-test.

Supplementary Figure S4

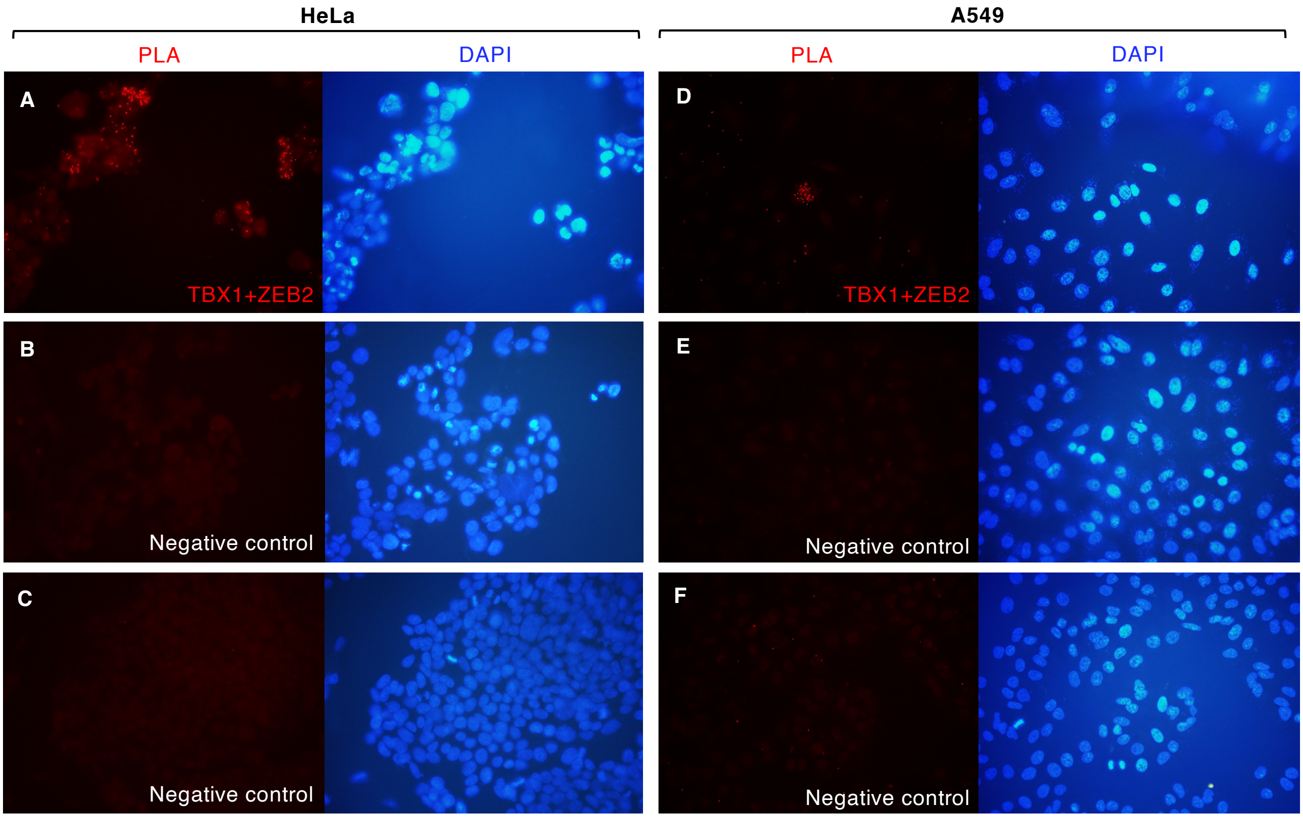

Supplementary Figure S4. *In situ* proximity ligation assay (PLA) detection of the TBX1 and ZEB2 colocalization.
(**A,D**) HeLa (**A**) and A549 cells (**D**) cotransfected with Myc-tagged TBX1 and Flag-tagged ZEB2. PLA shows a very close proximity between TBX1 and ZEB2, indicated by fluorescent spots (red).
(**B,E**) PLA without antibodies (assay negative control).
(**C,F**) PLA without transfection of constructs encoding TBX1 and ZEB2 (assay negative control).

Supplementary Figure S5

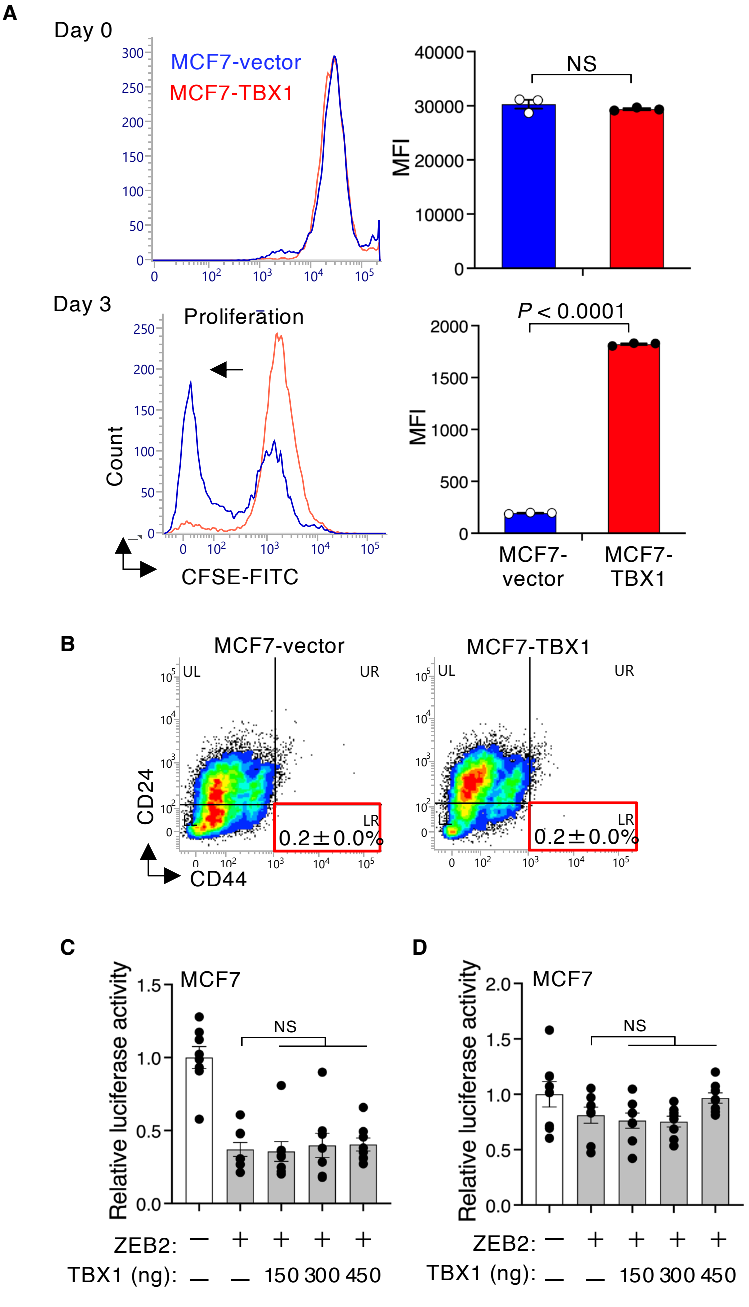

Supplementary Figure S5. TBX1 suppresses the proliferation of MCF7 cells.
(**A**) Flow cytometry analysis of empty vector and TBX1 stably transfected MCF7 cells labeled with the CFSE fluorescent dye: representative day 0 and day 3 histograms (left) and means ±s.e.m. of quantified results from three experiments with replicate wells (right) are shown.
(**B**) Flow cytometric analysis of CD24 and CD44 expression in empty vector (MCF7-vector) and TBX1 stably transfected MCF7 cells (MCF7-TBX1). The percentage of $CD44^{+}CD24^{-}$ stem cell population (lower right box) is indicated (n = 6).
(**C**) Relative luciferase activity in MCF7 cells transiently co-transfected with the *miR-200b-200a-429* promoter construct (-321/+120) , ZEB2 (150 ng), and the indicated amounts of TBX1 (n = 8).
(**D**) Relative luciferase activity in MCF7 cells transiently co-transfected with the *miR-203* promoter construct (-667/+77) , ZEB2 (150 ng), and the indicated amounts of TBX1 (n = 8).
For **A**, **C**, and **D**, the results are presented as mean ± s.e.m.; NS, not significant; unpaired two-tailed Student's t-test.

Supplementary Figure S6

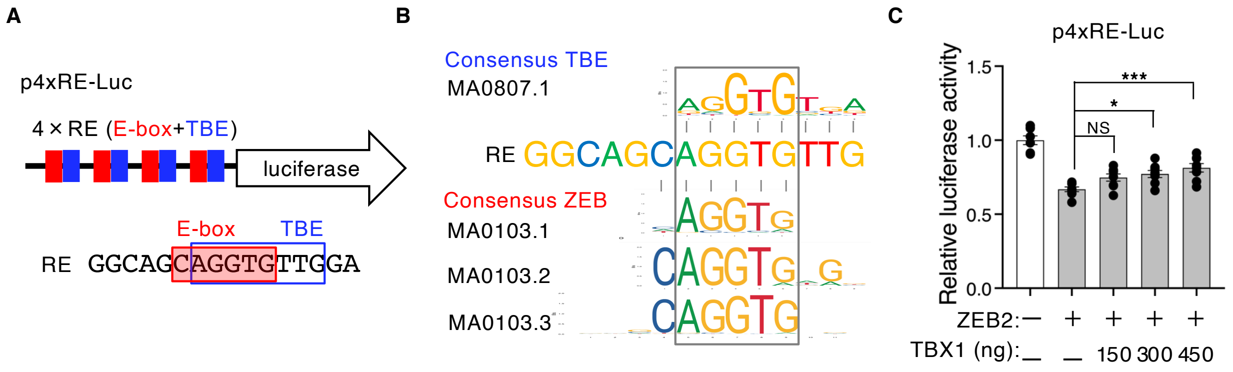

Supplementary Figure S6. TBX1 hardly affects the ZEB2-dependent inhibition of p4xRE-Luc.
(**A**) Schematic of the luciferase reporter plasmid (p4xRE-Luc) containing four copies of E-box and TBE.
(**B**) JASPAR sequence logos of E-box and TBE motifs. Box, overlapping core sequences.
(**C**) Relative luciferase activity in HeLa cells transiently co-transfected with p4xRE-Luc reporter (150 ng), ZEB2 (150 ng), and the indicated amounts of TBX1 (n = 8, $*P \leq 0.05$; $***P \leq 0.001$; NS, not significant; one-way ANOVA).

Supplementary Figure S7

A

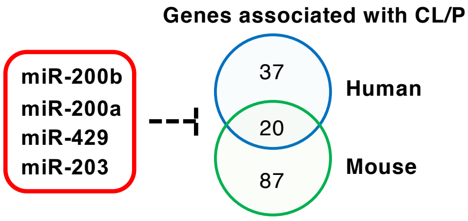

B

Species	miRNA	Predicted target genes associated with CL/P
**Human**	***hsa-mir-200a-3p***	*ASXL1 CDON FZD4 MBTPS2 NIPBL PLCB4 PTCH1 SLC35D1 TFAP2A TGFB2 TMEM216 TTC37* ***ZEB2***
	hsa-mir-200a-5p ***hsa-mir-200b-5p***	*ALX1 ARX BMP2 BRIP1 CHD7 DDX59 FAF1 FGFR1 FLRT3 FOXE1 FZD4 IMPAD1 KMT2D MYH9 NEBL PIEZO2 SLC39A13 SMS TBX22 TGFB2 TGFBR1 TGFBR2 TGIF1 TP63 TTC37* ***ZEB2***
	hsa-mir-200b-3p ***hsa-mir-429***	*CASK GABRB3 GLI3 GNAI3 IMPAD1 NIPBL NOG PTCH1 PTDSS1 PVRL1 SOX2 TFAP2A* ***ZEB2***
	hsa-mir-203a-3p	*ARID1B ASXL1 BMP2 CASK CTCF DLX5 EDN1 FLRT3 GABRB3 GLI3 GRHL3 MAFB PDGFRA PTCH1 PTDSS1 RPGRIP1L RUNX2 SHH SIX3 SMAD3 TGFB2 TGFB3 TGFBR2 TMEM67* ***ZEB2***
**Mouse**	***mmu-mir-200a-3p***	*Asxl1 Bnc2 Cdc42 Cdon Cyp26b1 Foxn3 Grb2 Hs2st1 Jag1 Kif3a Mapk1 Pbx1 Pds5b Pitx2 Prickle1 Ptch1 Rad23b Schip1 Serpinh1 Sox11 Sox5 Tcf21 Tfap2a Tgfb2 Tgfbr1* ***Zeb1***
	mmu-mir-200a-5p ***mmu-mir-200b-5p***	*Alx1 Alx4 Anp32 Bapaf1 Bmp2 Bmpr1a Bnc2 Cdon Chd7 Ctnnbip1 Dlx1 Ermp1 Ext1 Eya4 Fzd2 Golgb1 Gpc6 Gskip Ick Impad1 Irf6 Lrp6 Luzp1 Map3k7 Mdm4 Mef2c Mmp16 Oca2 Pds5b Robo1 Rspo2 Sgpl1 Sos1 Sox5 Sp8 Spry1 Tbc1d32 Tgfb2 Twist1 Whsc1 Wnt5a*
	mmu-mir-200b-3p ***mmu-mir-429-3p***	*Adamts3 Apaf1 Bmi1 Bnc2 Cask Cited2 Crebbp Crk Efna5 Efnb2 Fbxw7 Fgf10 Fign Glg1 Gli3 Hs2st1 Ick Impad1 Inhba Jag2 Mmp16 Pcsk5 Pds5b Piga Prdm16 Ror2 Schip1 Sh3pxd2a Smad7 Snai2 Sox5 Tfap2a* ***Vegfa Zeb1***
	mmu-mir-203-3p	*Acvr2a Adamts3 Ap2b1 Axin1 Bmi1 Bmp2 Bnc2 Cask Cited2 Crk Dlx5 Edn1 Ephb2 Ermp1 Ext1 Eya4 Fbxo11 Fign Gabrb3 Gbx2 Gli3 Grhl3 Gskip Hand2 Ick Inhba Kat6a Kcnj13 Lasp1 Mef2c Pdgfra Pitx2 Robo1 Ror2 Runx2 Shh Slc32a1 Snai2 Tgfb3* ***Vegfa*** *Wls Zmynd11*

Supplementary Figure S7. miR-200 and miR-203 predicted target genes associated with CL/P.
(**A**) Venn diagram depicting the overlap of human and mouse genes associated with CL/P. The numbers in each area represent the number of miR-200 and miR-203 predicted target genes in **B**.
(**B**) miR-200 and miR-203 predicted target genes associated with CL/P in humans and mice.

Supplementary Figure S8

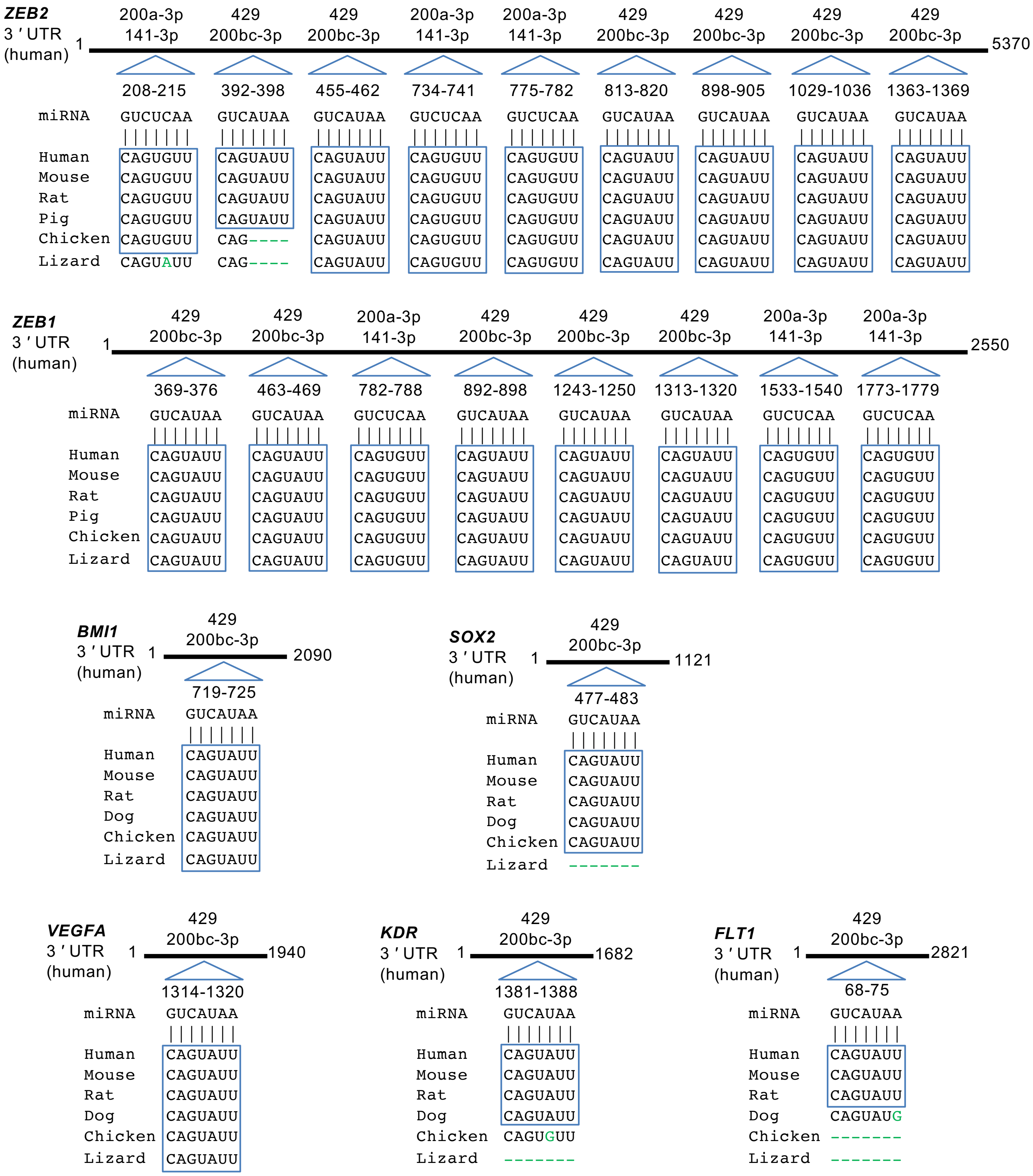

Supplementary Figure S8. A schematic diagram of binding sites for miR-200 predicted by TargetScan.
Blue boxes highlight sequence conservation among multiple species.

Supplementary Figure S9

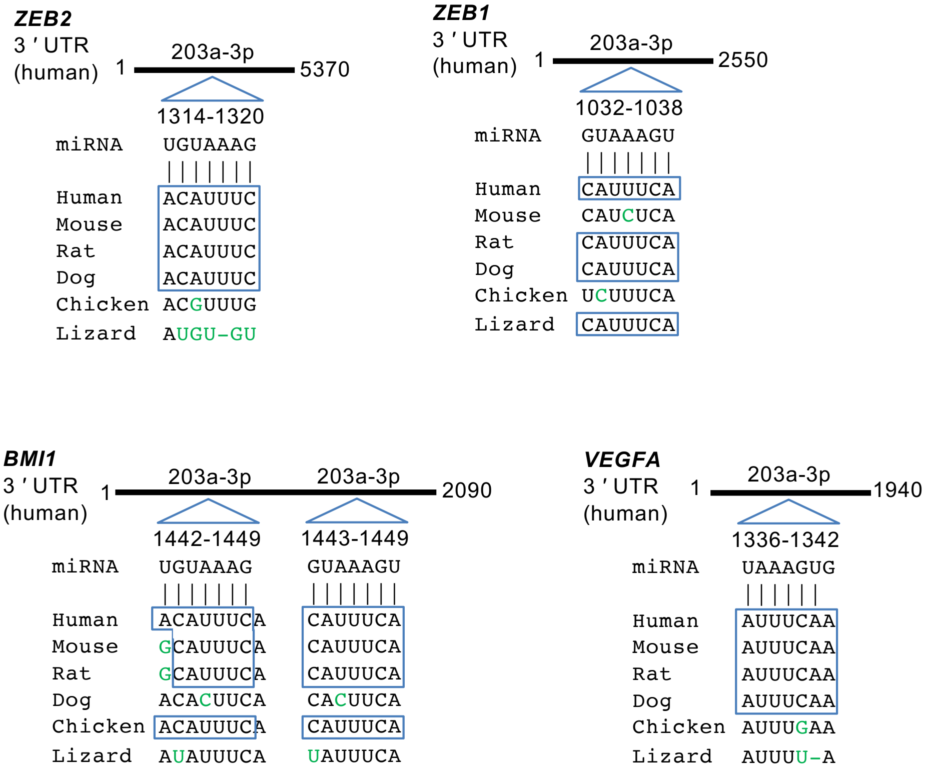

Supplementary Figure S9. A schematic diagram of binding sites for miR-203 predicted by TargetScan. Blue boxes highlight sequence conservation among multiple species.

Supplementary Figure S10

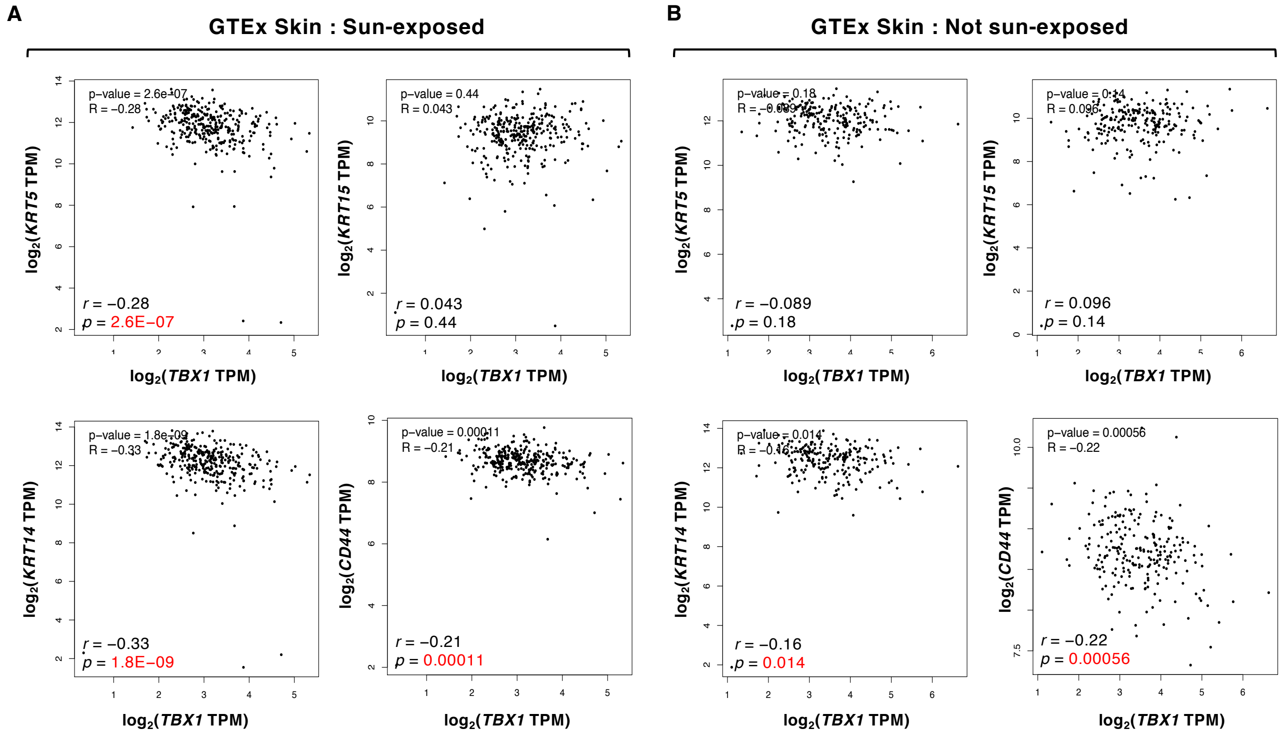

C

Skin		Sun-exposed			Not sun-exposed		
**Gene**	**Protein**	***r* (Pearson)**	***p*-value**	***p*-value summary**	***r* (Pearson)**	***p*-value**	***p*-value summary**
KRT5	keratin 5	−0.28	2.6E−07	****	−0.089	0.18	NS
KRT15	keratin 15	0.043	0.44	NS	0.096	0.14	NS
KRT14	keratin 14	−0.33	1.8E−09	****	−0.16	0.014	*
CD44	CD44 molecule	−0.21	0.00011	****	−0.22	0.00056	***

Supplementary Figure S10. Gene expression correlation between *TBX1* and basal cell markers.
(**A-C**) Correlation between the mRNA expression of *TBX1* and basal cell-specific genes in (**A**) sun-exposed (lower leg, n = 701) and (**B**) unexposed (suprapubic, n = 604) skin tissues.
(**C**) Table summarizing the results in **A, B**. Significant negative correlations between the mRNA expression levels of *TBX1* and basal cell markers are indicated in red. *$p \leq 0.05$, *** $p \leq 0.001$, **** $p \leq 0.0001$; NS, not significant.

Supplementary Figure S11

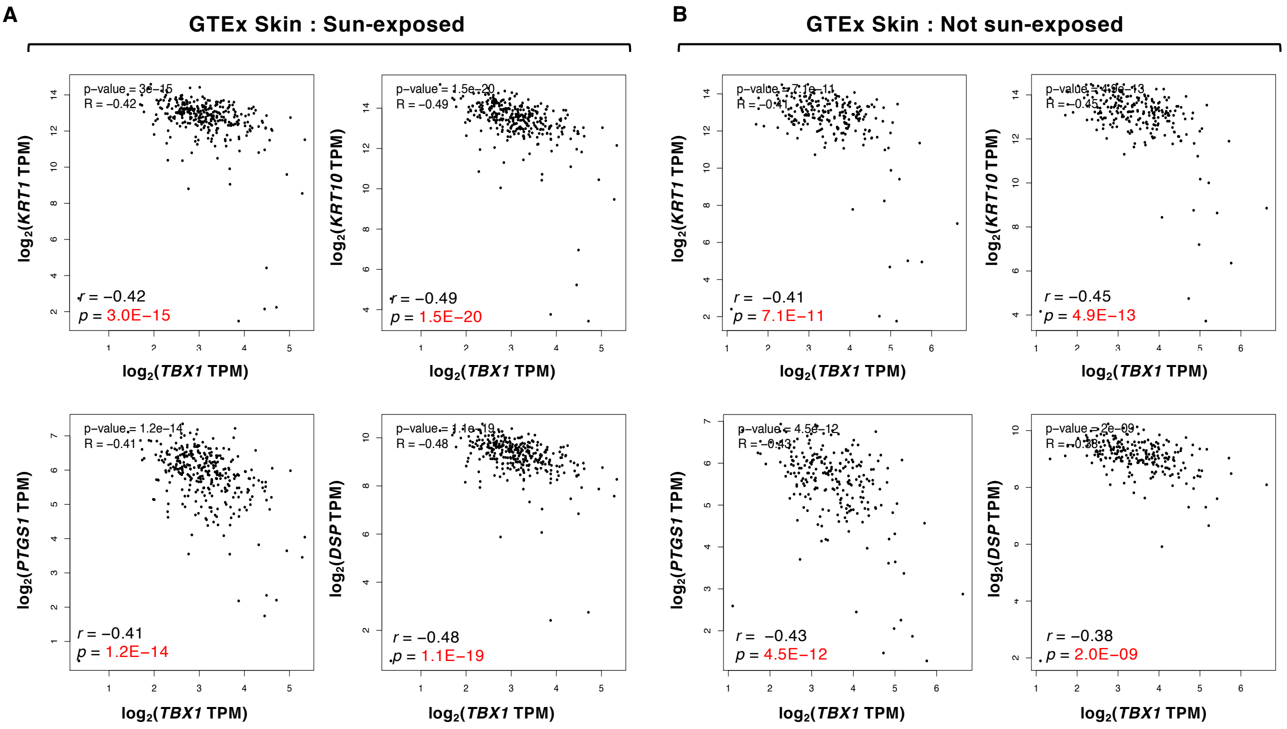

C

Skin		Sun-exposed				Not sun-exposed		
Gene	Protein	*r* (Pearson)	*p*-value	*p*-value summary		*r* (Pearson)	*p*-value	*p*-value summary
KRT1	keratin 1	−0.42	3.0E−15	****		−0.41	7.1E−11	****
KRT10	keratin 10	−0.49	1.5E−20	****		−0.45	4.9E−13	****
PTGS1	prostaglandin-endoperoxide synthase 1	−0.41	1.2E−14	****		−0.43	4.5E−12	****
DSP	desmoplakin	−0.48	1.1E−19	****		−0.38	2.0E−09	****

Supplementary Figure S11. Gene expression correlation between *TBX1* and suprabasal cell markers.
(**A-C**) Pearson correlation coefficient between mRNA expression of *TBX1* and suprabasal cell markers in (**A**) sun-exposed (lower leg) and (**B**) unexposed (suprapubic) skin tissue.
(**C**) Table summarizing the results in **A, B**. Significant negative correlations between mRNA expression levels of *TBX1* and suprabasal cell markers are indicated in red. ****$p \leq 0.0001$.

Supplementary Figure S12

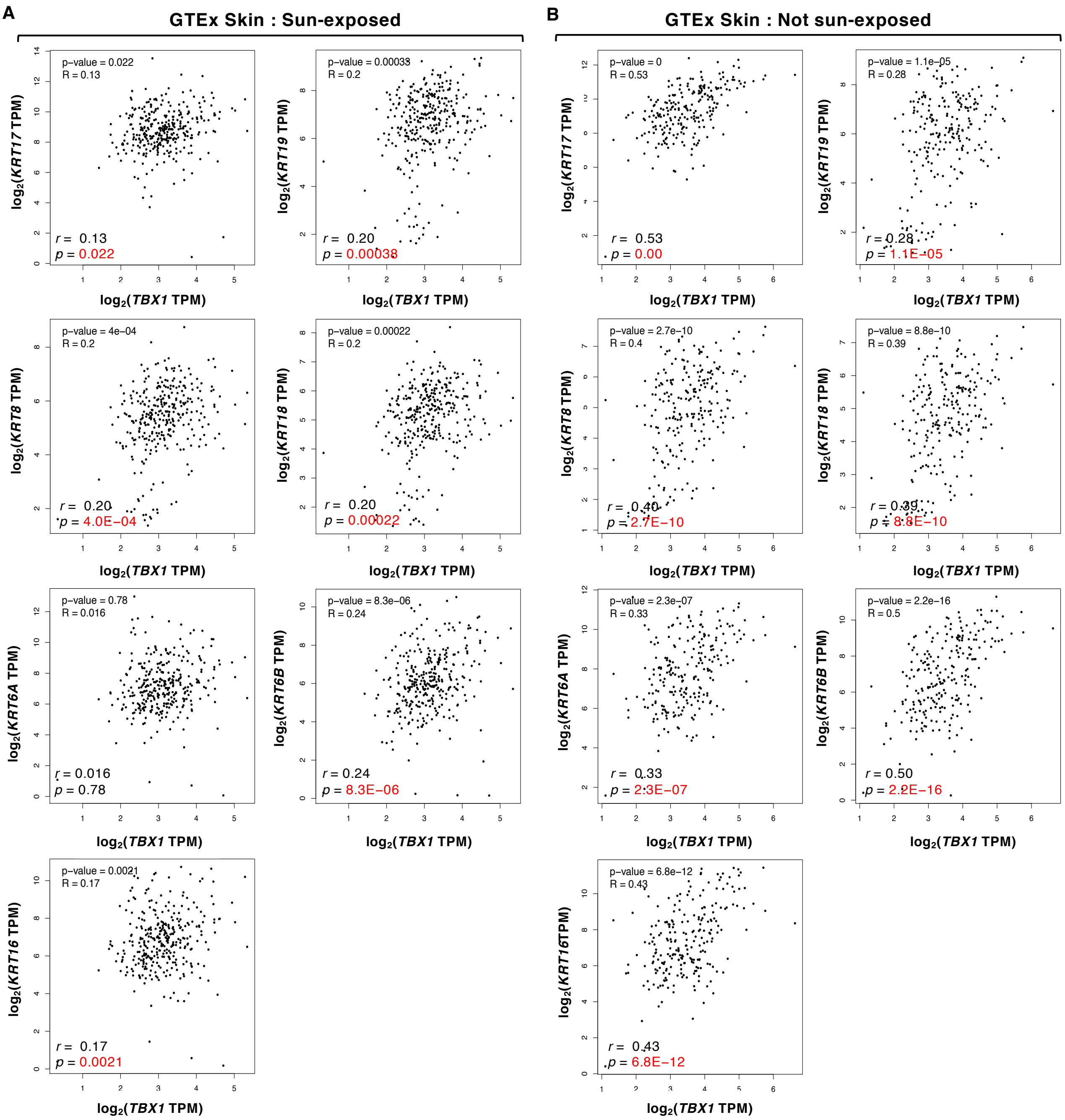

C

Skin		Sun-exposed			Not sun-exposed		
Gene	Protein	*r* (Pearson)	*p*-value	*p*-value summary	*r* (Pearson)	*p*-value	*p*-value summary
KRT17	keratin 17	0.13	0.022	*	0.53	0.00	****
KRT19	keratin 19	0.20	0.00038	***	0.28	1.1E−05	****
KRT8	keratin 8	0.20	4.0E−04	****	0.40	2.7E−10	****
KRT18	keratin 18	0.20	0.00022	***	0.39	8.8E−10	****
KRT6A	keratin 6A	0.016	0.78	NS	0.33	2.3E−07	****
KRT6B	keratin 6B	0.24	8.3E−06	****	0.50	2.2E−16	****
KRT16	keratin 16	0.17	0.0021	**	0.43	6.8E−12	****

Supplementary Figure S12. Gene expression correlation between *TBX1* and keratin markers
(**A-C**) Pearson correlation coefficient between mRNA expression of *TBX1* and keratin markers in (**A**) sun-exposed (lower leg) and (**B**) unexposed (suprapubic) skin tissue.
(**C**) Table summarizing the results in **A, B**. Significant positive correlations between mRNA expression levels of *TBX1* and keratin markers are indicated in red. *$p \leq 0.05$, **$p \leq 0.01$, ***$p \leq 0.001$, ****$p \leq 0.0001$; NS, not significant.

Supplementary Figure S13

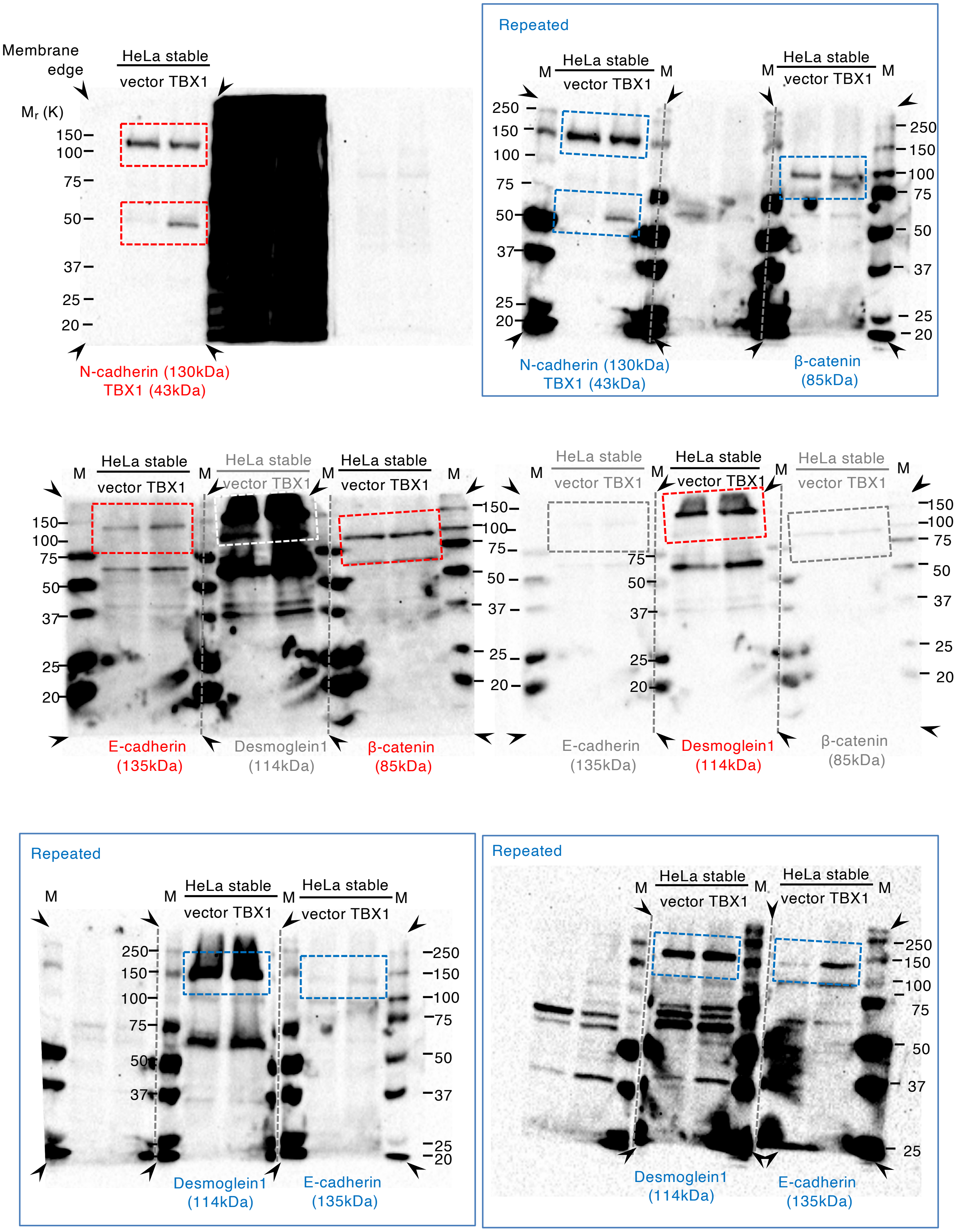

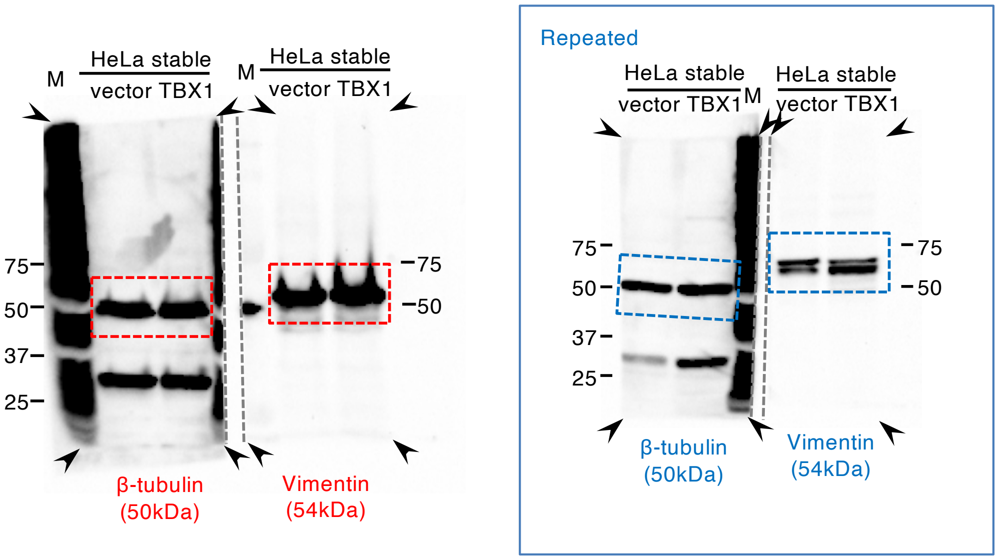

Supplementary Figure S13. Full-length images of representative Western blots related to Figure 1G. Images of selected portions are highlighted in red boxes. Arrowheads indicate membrane edges. M, Precision Plus Protein™ Dual Color Standards (Bio-Rad).

Supplementary Figure S14

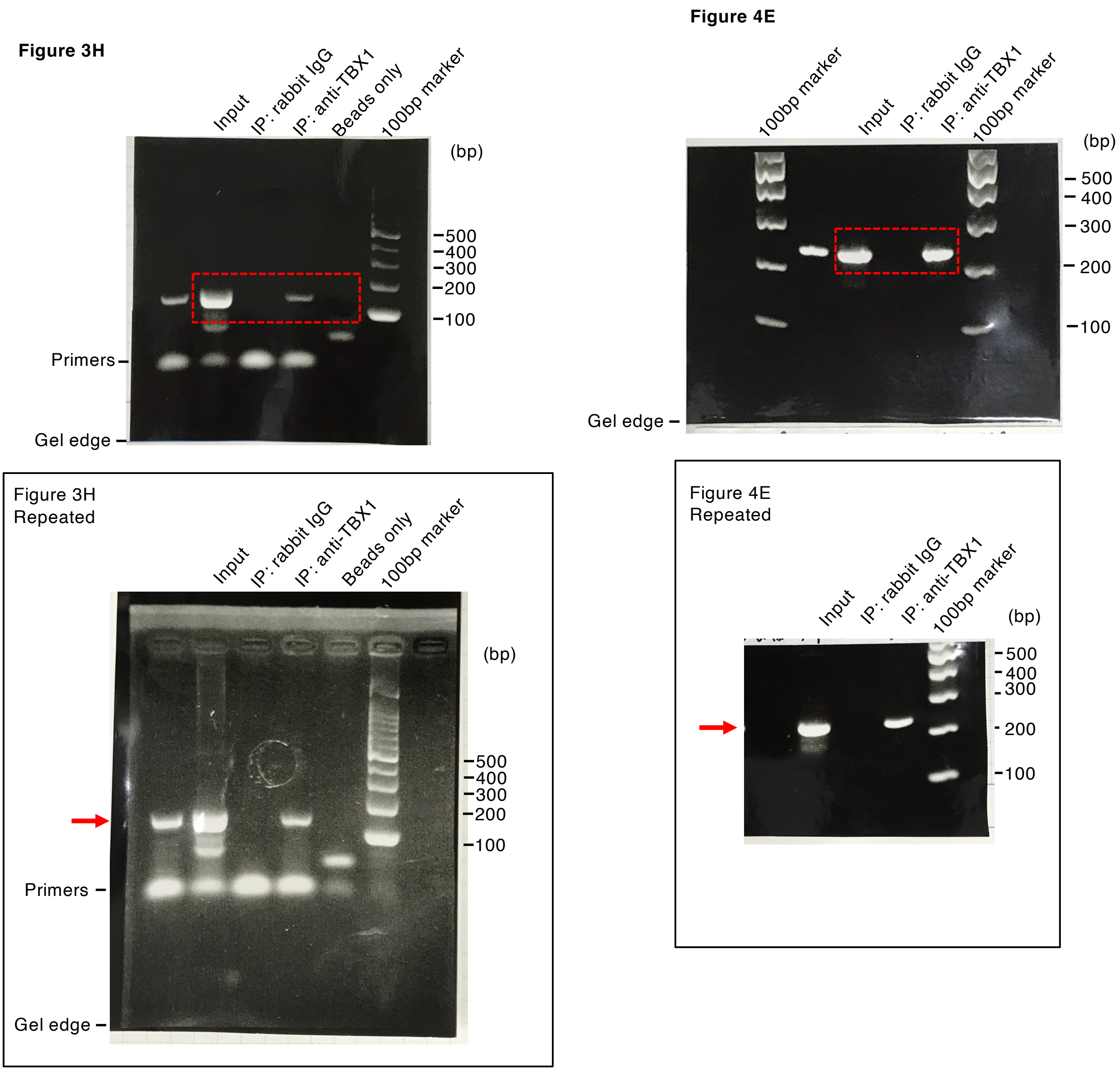

Supplementary Figure S14. Full-length images of representative ChIP assays related to Figures 3H and 4E.
Images of selected portions are highlighted in red boxes.
